# Sam-Sam Association Between EphA2 and SASH1: In Silico Studies of Cancer-Linked Mutations

**DOI:** 10.3390/molecules30030718

**Published:** 2025-02-05

**Authors:** Marian Vincenzi, Flavia Anna Mercurio, Ida Autiero, Marilisa Leone

**Affiliations:** Institute of Biostructures and Bioimaging, National Research Council of Italy, Via Pietro Castellino 111, 80131 Naples, Italy; marian.vincenzi@ibb.cnr.it (M.V.); flaviaanna.mercurio@cnr.it (F.A.M.); ida.autiero@cnr.it (I.A.)

**Keywords:** EphA2, SASH1, cancer, missense mutation, Sam-Sam complex, Mid-Loop (ML)/End-Helix (EH) model, structure prediction, docking, molecular dynamics

## Abstract

Recently, SASH1 has emerged as a novel protein interactor of a few Eph tyrosine kinase receptors like EphA2. These interactions involve the first N-terminal Sam (sterile alpha motif) domain of SASH1 (SASH1-Sam1) and the Sam domain of Eph receptors. Currently, the functional meaning of the SASH1-Sam1/EphA2-Sam complex is unknown, but EphA2 is a well-established and crucial player in cancer onset and progression. Thus, herein, to investigate a possible correlation between the formation of the SASH1-Sam1/EphA2-Sam complex and EphA2 activity in cancer, cancer-linked mutations in SASH1-Sam1 were deeply analyzed. Our research plan relied first on searching the COSMIC database for cancer-related SASH1 variants carrying missense mutations in the Sam1 domain and then, through a variety of bioinformatic tools and molecular dynamic simulations, studying how these mutations could affect the stability of SASH1-Sam1 alone, leading eventually to a defective fold. Next, through docking studies, with the support of AlphaFold2 structure predictions, we investigated if/how mutations in SASH1-Sam1 could affect binding to EphA2-Sam. Our study, apart from presenting a solid multistep research protocol to analyze structural consequences related to cancer-associated protein variants with the support of cutting-edge artificial intelligence tools, suggests a few mutations that could more likely modulate the interaction between SASH1-Sam1 and EphA2-Sam.

## 1. Introduction

SASH1 (SAM and SH3 domain containing 1) is an adaptor protein that belongs to the Sly (Src homology domain 3 lymphocyte protein)/SASH1 group. This protein family includes three highly homologous members (Sly1, Sly2, and SASH1), all sharing within their domain arrangement a bipartite nuclear localization signal, an SH3 (Src Homology 3), and a Sam (Sterile alpha motif) domain. SASH1, differently from Sly1 and Sly2, also presents at the N-terminus a coiled-coil domain and includes two Sam domains instead of a single one. SASH1 was originally described in 2003 and linked to potential tumor suppressor activity in breast cancer [1]. Different roles have been attributed to SASH1, for example, it has been suggested that there is a positive correlation between expression levels of SASH1 and enhanced apoptosis [1].

SASH1 has been related to diverse pathological conditions, including atherosclerosis and dermatological pathologies, but it has been widely investigated in the framework of human cancer [1]. SASH1 downregulation in different cancer types correlates with decreased patient survival and enhanced formation of metastasis, probably due to its pro-apoptotic activity and the capacity to block EMT (epithelial–mesenchymal transition). In breast cancer tissues, a significant downregulation of SASH1 expression has been observed, whereas upregulation of SASH1 is able to block the proliferation and invasion of breast cancer cells through inhibition of the PI3K-Akt-mTOR (mammalian target of rapamycin) signaling route [2]. In adenocarcinoma, little SASH1 mRNA expression is linked to poor survival. Moreover, following treatment of lung cancer cells with a molecule (i.e., chloropyramine) that increases SASH1 protein levels, decreased cell proliferation, and enhanced sensitivity to cisplatin can be observed [3].

Several interactors of SASH1 have been reported in the literature, including the TNF receptor-associated factor 6 (TRAF6), IκB kinase α, IκB kinase β, cortactin, and CRKL (CT10 regulator of kinase-like) [1].

**Figure 1:**
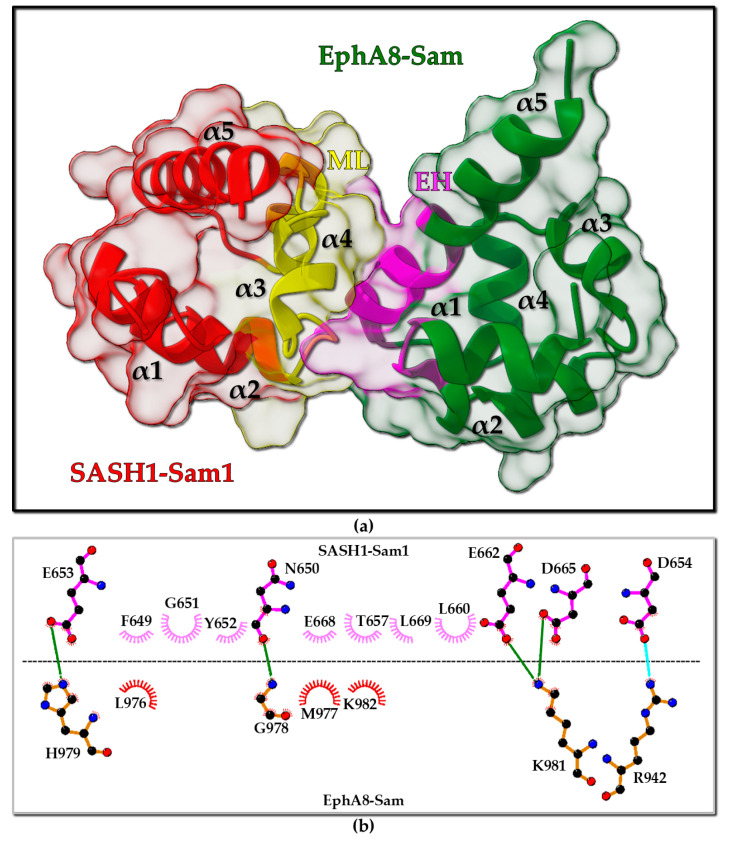
(**a**) X-ray structure of Mus musculus SASH1-Sam1 (UniProtKB [30] entry P59808 residues P625-Y690) in complex with Mus musculus EphA8-Sam (UniProtKB [30] entry O09127 residues L932–S996) (pdb entry 8J1I [9]). SASH1-Sam1 is reported in red with the ML region (residues F649-L669) colored yellow. Dark green and magenta highlight EphA8-Sam and its EH region (residues I941-M943 and M977-G985), respectively. (**b**) LigPlot+ [11,31] analysis of the interaction interface: black, blue, and red spheres refer to carbon, nitrogen, and oxygen atoms, respectively; green and cyan solid lines refer to H-bonds and salt bridges, respectively; non-bonded interactions are represented by the crescents with bristles.

As mentioned above, SASH1 contains two Sam domains within its protein domain organization. Sam domains are protein interaction modules with a five-helix bundle fold (Figure 1a) able to bind a variety of partners like other proteins, RNA, lipids but that also are known to self-associate via homotypic and heterotypic interactions to form small oligomers or polymers [4,5]. Sam domains are very versatile, as they are able to modulate several protein–protein interactions, including those involved in cancer development and metastasis [6]. An interesting review lately described how multi-Sam domain-containing proteins can be involved in several cancer-related activities [6]. Recently, novel interactions associated with the first N-terminal Sam domain of SASH1 (SASH1-Sam1) have been reported. SASH1 has been identified as a novel binding partner of Caskin1 (CASK (calcium/calmodulin-dependent serine protein kinase) interacting protein 1), a protein that contains two tandem Sam domains. AlphaFold2 (AF2) [7] structure predictions coupled to mutagenesis studies suggested a Mid-Loop (ML)/End-Helix (EH) interaction mode for the Sam–Sam interaction between SASH1-Sam1 and Sam domains from Caskin1; this interaction also appears to interfere with Caskin1 polymerization mediated by the Sam domains [8]. Moreover, SASH1 has emerged as a novel protein interactor of a few Eph tyrosine kinase receptors like EphA8 and EphA2 while being unable to interact with EphA4 through a yeast two-hybrid screening using mouse proteins [9]. Interactions between SASH1 and Eph receptors involve the first N-terminal Sam domain of SASH1 (SASH1-Sam1) and the Sam domain of Eph receptors (Eph-Sam). The mouse SASH1-Sam1/EphA8-Sam complex has been very well characterized. A protein construct including the SH3 and the Sam1 domains of mouse SASH1 is able to bind EphA8-Sam with a dissociation constant in ITC (isothermal titration calorimetry) studies equal to 1.09 µM, that is very similar to what observed for a SASH1-Sam1 sample, including only the Sam1 domain (K_D_ = 0.88 µM) [9]. Structural studies by X-ray crystallography indicate that SASH1-Sam1 associates with EphA8-Sam by forming a dimer with the ML/EH binding topology (Figure 1a). The ML/EH model is adopted by most Sam–Sam complexes [4,5,10]. SASH1-Sam1 provides the ML binding site with its central region, whereas the C-terminal helix α5 in EphA8-Sam and the α1α2 loop contribute to the EH interaction region (Figure 1a). The complex is stabilized by many electrostatic interactions, as clearly shown by the diagram of intermolecular contacts generated with LigPlot+ [11] (Figure 1b). The SASH1-Sam ML site is negatively charged and includes several key residues (i.e., E653, D654, D656, E662, and D665) that provide salt bridges/H-bond contacts with positively charged residues positioned inside the EphA8-Sam EH surface (i.e., R942, H979, and K981) (Figure 1b). In addition to the interactions shown by LigPlot+ analysis, a salt bridge between R942 (EphA8) and D656 (SASH1) stabilizes the ML/EH association between SASH1-Sam1 and EphA8-Sam [9]. LigPlot+ is possibly unable to detect this important interaction due to the double conformation of D656 within the X-ray structure [9]. Indeed, a D656A SASH1-Sam1 mutant fails to bind EphA8-Sam. Another important residue is G978 in EphA8-Sam that forms a backbone–backbone intermolecular H-bond with N650 in SASH1-Sam1 and makes crucial contacts with Y652 (Figure 1b). The presence of a glycine at the N-terminus of the α5 helix is a characteristic of Sam domains participating to Sam–Sam interactions via the EH interface, as it provides low steric hindrance and an anchorage point through pivotal intermolecular contacts for the ML interface [9,12,13]. Interestingly, the SASH1-Sam1 Y652A mutation inhibits the formation of the SASH1-Sam1/EphA8-Sam complex, whereas the leukemia-linked mutation G978D in EphA8-Sam highly reduces the binding affinity for SASH1-Sam1 by interfering with the network of interactions engaging the key G978 residue. Remarkably, another cancer-related mutation in EphA8-Sam (i.e., the melanoma-linked R942H substitution) also blocks the interaction with SASH1-Sam1 by possibly avoiding the formation of crucial contacts with D656 from SASH1-Sam1.

From the functional point of view, the association between EphA8-Sam and SASH1-Sam1 appears important to regulate EphA8 kinase activity [9].

Yeast two-hybrid screening also pointed out a strong interaction between the EphA2 receptor and SASH1-Sam1 (K_D_ = 2.52 µM using a SASH1 mouse construct, including the SH3 and Sam1 domains) [9].

The EphA2 receptor is also a key player in cancer and is upregulated in many tumor types, reaching the highest levels in the cancer cells with the largest degree of invasiveness [14]. EphA2 upregulation in tumors is usually associated with disease progression and malignancy; this receptor has also been implicated as a key mediator of breast cancer EMT and metastasis [15]. Similarly, EphA2 might also be linked to GBM (glioblastoma) as a regulator of tumorigenesis, invasion, metastasis, and angiogenesis [16]. EphA2 signaling is very complex and can control cellular activities in different and conflicting ways [17]. EphA2 overall effect in cancer cells derives from a fine balance between the mainly antioncogenic, ephrin ligand-dependent canonical pathway and the uncanonical pathway that does not rely on ephrin ligand binding and is mostly considered pro-oncogenic [18,19].

EphA2 has a C-terminal Sam domain (EphA2-Sam) that binds to several partners, including the Sam domain of the lipid phosphatase Ship2 (SH2-containing 5′-inositol phosphatase 2) (Ship2-Sam) [20,21] and the first Sam domain of the adaptor protein Odin [22]. The role of the Sam domain within receptor signaling has started to be clarified only lately. EphA2-Sam has been connected to decreased receptor oligomerization and activation of the kinase activity [23,24]. EphA2-Sam acts as a negative modulator not only for the ligand-independent EphA2 oligomerization but also can lower receptor clustering upon binding to the ephrin ligand [23].

It has been suggested that the Sam domain of EphA2 could indeed inhibit receptor oligomerization and thus influence kinase activity in the absence of a ligand by either binding to the kinase domain or through interaction with other partner Sam domains [24].

The interaction between EphA2-Sam and Ship2-Sam has been very well characterized from a structural and functional point of view [20,21,25,26]. First of all, Ship2 is a negative modulator of receptor endocytosis [26] and its association through Sam–Sam interaction with EphA2 is likely connected to pro-cancer effects in cancer cells, as it also favors EphA2 ligand-independent pro-migratory roles [21].

The Ship2-Sam/EphA2-Sam complex (K_D_ equal to 0.75 ± 0.12 µM) has an ML/EH interaction topology where Ship2-Sam forms the ML interface, which is rich in negatively charged residues, while EphA2-Sam forms a positively charged EH interface [21]. A Gly residue at the N-terminal side of the α5 helix inside the EphA2-Sam EH interface is important to provide intermolecular contacts with the Ship2-Sam ML interface [12]. Structural features of the human Ship2-Sam/EphA2-Sam complex closely resemble those observed in the crystal structure of the mouse SASH1-Sam1/EphA8-Sam complex, and it is plausible to speculate that a similar ML/EH complex will arise when SASH1-Sam1 interacts with EphA2-Sam. To date, the SASH1-Sam1/EphA2-Sam complex has not been characterized, and especially its functional meaning is unknown. Due to the well-established correlation of both EphA2 and SASH1 with tumor onset and progression, herein, to investigate how/if the formation of the SASH1-Sam1/EphA2-Sam complex could affect EphA2 activities in cancer, we focused on cancer-linked mutations in SASH1-Sam1. We adopted and refined a protocol that has already been implemented in our laboratory to study cancer-related mutations in the context of the EphA2-Sam/Ship2-Sam complex [21]. Our research plan relies first on searching the COSMIC (Catalogue of Somatic Mutations in Cancer) [27] database for cancer-related SASH1 variants carrying missense mutations in the Sam1 domain and then, through several bioinformatic tools and molecular dynamic simulations, studying how these mutations could affect the stability of SASH1-Sam1 alone, leading eventually to a defective fold. Next, through docking studies, with the support of Haddock [28] and AF2 [7,29] structure predictions, we investigate if/how cancer-linked mutations in SASH1-Sam1 could affect binding to EphA2-Sam. This study not only proposes an efficient protocol to investigate structural consequences related to cancer-associated protein variants with the support of cutting-edge artificial intelligence tools (i.e., AF2) but also highlights a few mutations that could more likely negatively modulate the binding of SASH1-Sam1 to EphA2-Sam and that can be thus prioritized in experimental studies.

## 2. Results and Discussion

**Figure 2:**
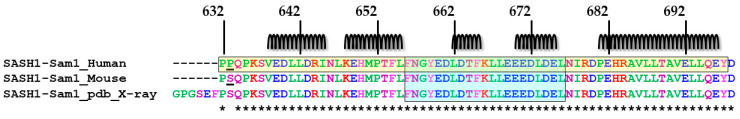
Alignment of the human SASH1 sequence (residue range P632-D698 encompassing the Sam1 domain (P633-Y697)), the corresponding sequences of SASH1 from Mus musculus (residues P625-D691, encompassing the Sam1 domain (S626-Y690)), and that of the X-ray structure [9]. Secondary structure elements are reported on top. Different residues in human and mouse sequences have been underlined. The ML interfaces are enclosed within the cyan rectangle. The sequence from human SASH1-Sam1 used to generate the AF2 models is highlighted by a yellow rectangle. Further details are given in the Materials and Methods section.

**Figure 3:**
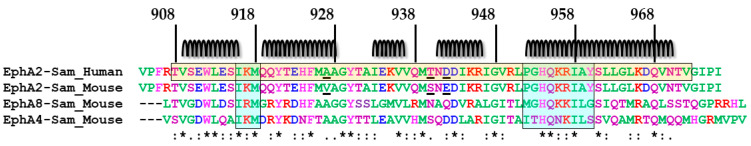
Alignment of the human EphA2 sequence (residue range V904-I976 encompassing the Sam domain (V904-Q968)), the corresponding sequences of Mouse EphA2 (residues V905-I977, encompassing the Sam domain (V905-Q969)), Mouse EphA8 (residues L932-L1004, covering most of the Sam domain (N929-Q993)), and Mouse EphA4 (residues V914-V986, encompassing most of the Sam domain (S911-Q975)). The EH interfaces are enclosed within the cyan rectangle. The sequence from human EphA2-Sam used to generate the AF2 models is highlighted by a yellow rectangle. Different amino acids between human and mouse EphA2-Sam sequences have been underlined. Further details are given in the Materials and Methods section.

Before starting to select cancer-related mutations within the SASH1-Sam1 domain, we deeply analyzed and compared the primary sequences of SASH1 and several Ephs receptors (Figure 2 and Figure 3) from different organisms (i.e., Homo sapiens and Mus musculus). This analysis was necessary as previous functional and structural studies were conducted on the SASH1-Sam1/EphA8-Sam complex from Mus musculus, while we are interested in analyzing the human SASH1-Sam1/EphA2-Sam interaction.

Interestingly, mouse and human SASH1-Sam1 primary sequences are almost identical with only one N-terminal amino acid residue difference (Figure 2).

Next, we carried out a comparison of amino acid sequences between EphA2-Sam (from Mus musculus and Homo sapiens) and either EphA8-Sam (mouse sequence), which has a similar binding affinity for SASH1-Sam1, or EphA4-Sam (mouse sequence), which is instead unable to bind SASH1-Sam1 (Figure 3) [9]. To assess sequence identity, we employed the SIM [32]—Alignment Tool for Protein Sequences in EXPASY [33] (https://web.expasy.org/sim/, access data 13 December 2024) (Figure S1). Mouse and human EphA2-Sam present very high homology with 95.9% identity considering 73 residues overlap; lower and similar sequence identities can be revealed between EphA2-Sam and EphA8-Sam (~38%) or EphA4-Sam (~43%) (Figure S1).

### 2.1. Cancer-Related Mutations in SASH1-Sam1

We looked for cancer-related mutations affecting SASH1-Sam1 in COSMIC (Catalogue of Somatic Mutations in Cancer) [27]. Results are shown in Table 1.

Interestingly, SASH1-Sam1 mutations are found in cancer tissues that can also be associated with EphA2 (Table 1). In fact, EphA2 levels are upregulated in many cancer types (i.e., gastric, esophageal, colorectal, cervical, breast, ovary, prostate, pancreas, neck, renal, lung cancers, melanoma, and glioblastoma [4,34,35]). In addition, EphA2 plays a role in the onset and progression of hepatocellular carcinoma [36] and has been largely linked to bladder cancer, representing one of the main urinary tract tumors [37,38].

**Figure 4:**
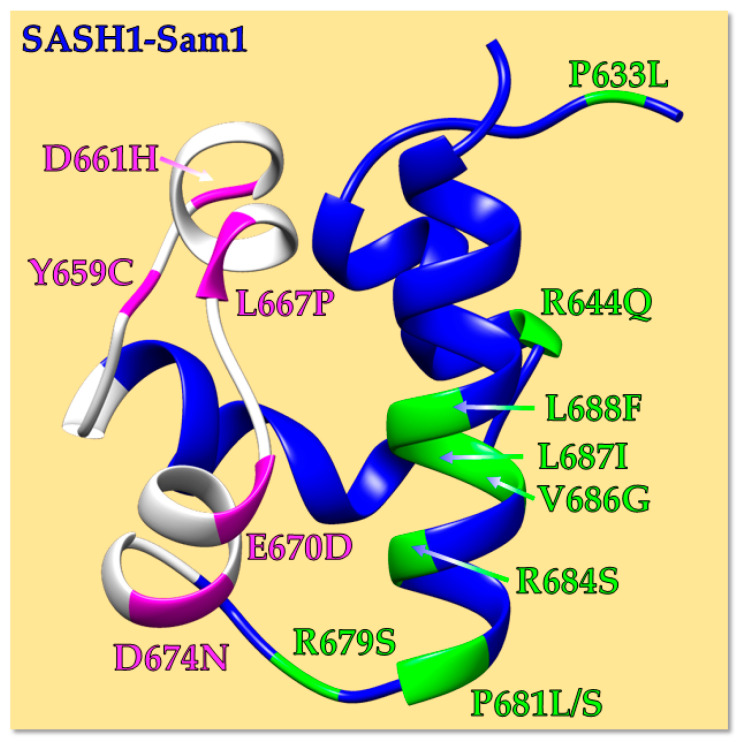
AF2 model [53,54] of human SASH1-Sam1 (blue) in a ribbon representation. The ML interface (residues from F656 to L676) is colored white, while diverse mutations are colored in green and magenta if positioned far away from or inside the ML, respectively.

In detail, fourteen SASH1 missense mutations could be identified, with 5/14 of them affecting amino acid residues positioned inside the ML interface of SASH1-Sam1 (Table 1 and Figure 4).

We next further checked all SASH1-Sam1 variants through AlphaMissense [39]. Similarly to AlphaFold, which predicts protein structures, AlphaMissense is an artificial intelligence-relying tool that predicts the pathogenicity of missense variants [39]. Interestingly, as expected, most of such mutations were predicted to be pathogenic; results are ambiguous for only three SASH1-Sam1 variants, including the D674N mutation that affects a residue positioned inside the ML interface (Table 1 and Figure 2 and Figure 4).

Conservation scores for diverse SASH1-Sam1 mutated residues were obtained by Consurf [40,41] (Table S1). The ConSurf scores are in the range 1–9; “1” stands for variable, whereas “9” for conserved amino acids. Intriguingly, mutations referring to amino acids positioned inside the ML binding site of SASH1-Sam1 (Table 1 and Figure 4) that are deputed to the interaction with the EH of Ephs-Sam (Figure 2 and Figure 3) are, except L667, rather well conserved (scores ≥ 5) and thus expected to have a large impact on protein structural and functional characteristics (Table S1).

In addition, residues affected by cancer-related mutations and positioned within the ML interface have good solvent exposure (>20% considering all backbone and side-chain atoms), except Y659, which appears more buried (Figure 4 and Table S1), and mutation of such residues could likely perturb more the SASH1-Sam1 fold, while the other mutations could more likely affect interactions with binding partners.

**Table 1:** List of missense mutations of the Sam1 domain from human SASH1 found in the COSMIC catalogue [27] (https://cancer.sanger.ac.uk/cosmic/gene/analysis?ln=SASH1, access date 12 September 2024). Mutations within the ML region (residues F656-L676) are underlined (I column). “Count” (II column) stands for the number of samples presenting the matching mutation. The type of tumor where the mutation has been identified is indicated as well (III column). SASH1 variants not associated with references derive from the Sanger Institute Cancer Genome Project or the ICGC/TCGA (International Cancer Genome Consortium/The Cancer Genome Atlas). The pathogenicity scores and consequent classes predicted by AlphaMissense for each mutation (range for likely benign = 0–0.34, range for ambiguous = 0.34–0.564, range for likely pathogenic (first level) = 0.564–0.78, and range for likely pathogenic (second level) = 0.78–1.0) are indicated in the IV column [42,43].

SASH1-Sam1 Mutations	Count	Tumor Location and Histology	AlphaMissense/ Pathogenicity Class
P633L ^@^	1	Skin (malignant melanoma)	0.1001/likely_benign
R644Q	1	Skin (carcinoma, basal cell carcinoma) [44]	0.5098/ambiguous
Y659C	2	Liver (carcinoma, hepatocellular carcinoma) Lung (carcinoma, adenocarcinoma) [45]	0.999/likely_pathogenic
D661H	1	Urinary tract (carcinoma, transitional cell carcinoma) [46]	0.992/likely_pathogenic
L667P	1	Esophagus (carcinoma, squamous cell carcinoma) [47]	0.9998/likely_pathogenic
E670D	1	Lung (carcinoma, non-small cell carcinoma) [48]	0.8412/likely_pathogenic
D674N	1	Skin (malignant melanoma) [49]	0.5289/ambiguous
R679S	1	Lung (carcinoma, small cell carcinoma) [50]	0.572/likely_pathogenic
P681L	1	Skin (malignant melanoma)	0.5354/ambiguous
P681S	1	Skin (malignant melanoma)	0.4545/ambiguous
R684S	1	Lung (carcinoma, adenocarcinoma)	0.9968/likely_pathogenic
V686G	1	Large intestine (carcinoma, adenocarcinoma)	0.9396/likely_pathogenic
L687I	1	Lung (carcinoma, adenocarcinoma) [51]	0.8305/likely_pathogenic
L688F	1	Large intestine (adenoma) [52]	0.9339/likely_pathogenic

^@^ Amino acid substitutions refer to the sequence numbering of the UniprotKB [30] entry O94885 for human SASH1.

### 2.2. Investigating SASH1-Sam1 Mutant Stability by Bioinformatic Tools

#### 2.2.1. Analysis of the Amino Acid Sequence: Chemical–Physical Properties

The Protparam tool in EXPASY [33,55] was employed to predict possible changes in the chemical–physical properties of SASH1-Sam1 induced by cancer-related mutations (Table 1 and Table S2). Due to the very high identity of SASH1-Sam1 sequences from Homo sapiens and Mus musculus (Figure 2), differences between their chemical–physical features are irrelevant.

The grand average of hydropathy (GRAVY) value for a protein refers to the sum of the hydropathy values of the single amino acid residues divided by the total number of residues [55]. Negative GRAVY values are associated with hydrophilic proteins, and positive values are associated with hydrophobic ones [56]. Based on GRAVY values, all SASH1-Sam1 variants can be defined as hydrophilic domains (Table S2). P to L substitutions (i.e., mutations P633L and P681L) induce the largest increase in hydrophobicity; the L667P mutation affecting the ML interface is associated with improved hydrophilicity (Figure 4 and Table S2).

Regarding the pI (isoelectric point) values, no major variations related to the mutations can be pointed out (Table S2).

The instability index (Table S2) relies on the presence within the primary sequence of specific dipeptide stretches and provides an estimate of the possible effects that a mutation can have on SASH1-Sam1 stability in vitro [55,57]. A stable protein should be characterized by an instability index smaller than 40 [55]. For all SASH1-Sam1 variants, the predicted instability indices are slightly higher than 40, and according to this parameter, the most destabilizing mutations should be Y659C (within the ML interface), P681S, and L687I (Table S2).

#### 2.2.2. Three-Dimensional (3D) Structural Models Generation and Analysis

Cancer-related missense mutations might affect the SASH1-Sam1 domain in the following two different ways: 1—by producing structural defects and/or 2—by hampering protein–protein interactions. To possibly understand structural variations that could be associated with pathological mutations and also disturb the SASH1-Sam1 interaction network, we built 3D models of SASH1-Sam1 native and cancer-related variants by just focusing on mutations included inside the ML binding site (Figure 2 and Figure 4).

**Figure 5:**
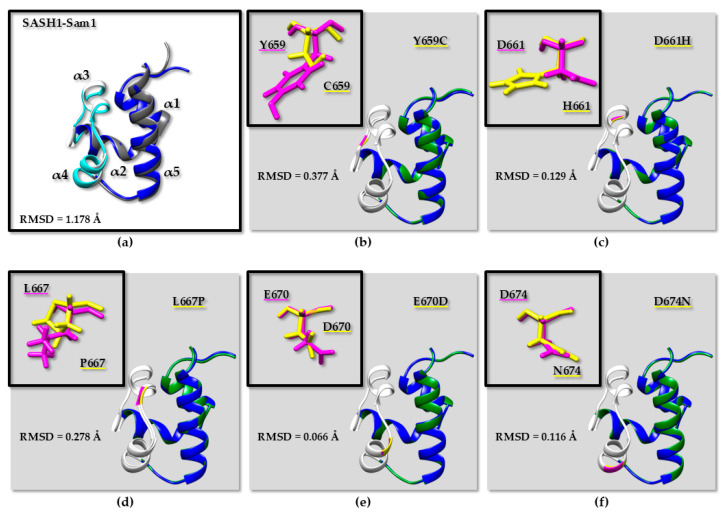
(**a**) Comparison of 3D coordinates of the best AF2 [53,54] model of wild-type human SASH1-Sam1 (WT, blue) (residue range P632-Y697) and those of the mouse SASH1-Sam1 X-ray structure (grey) (pdb entry code 8J1I [9], residues P625-Y690). Backbone atoms were overlayed to evaluate the indicated RMSD value. (**b**–**f**) Superpositions on the backbone atoms of AF2 [53,54] models of the human SASH1-Sam1 (blue) and diverse cancer-related mutants (green). Only comparisons between the wild-type human SASH1-Sam1 and cancer-related mutants, carrying amino acids substitutions positioned inside the ML interaction interface (residues F656-L676), were considered. Results for Y659C, D661H, L667P, E670D, and D674N mutations are shown in panels (**b**), (**c**), (**d**), (**e**), and (**f**), respectively. The ML interface is shown in white in all SASH1-Sam1 AF2 models (**a**–**f**) and in cyan in the X-ray structure (**b**–**f**). The upper left inserts in each panel feature superpositions between side chains of native residues (magenta) and mutated (yellow) amino acids.

The cutting-edge artificial intelligence tool AlphaFold2 (AF2) [53,54] was implemented to predict 3D structures of human SASH1-Sam1 domains in the native and mutated forms (Figure S2). A comparison of 3D coordinates by RMSD (Root Mean Square Deviation) evaluation (Tables S3 and S4 and Figure S2 and Figure 5) demonstrates no dramatic differences in the overall structure arrangement of human and mouse SASH1-Sam1 domains (Figure 5a) or human SASH1-Sam1 native and cancer-related variants (Figure 5b–f). Indeed, inspection of RMSD values (Table S3) indicates the largest variation when comparing our AF2 model of the human SASH1-Sam1 domain with the X-ray structure of mouse SASH1-Sam1 (Figure 5a and Table S3). This result is not surprising and could also be partially due to the different optimization protocols we employed to obtain the AF2 model with respect to the procedure used to generate the crystal structure of mouse SASH1-Sam1. However, as clear in Figure S2a,b and Figure 5a, the overall fold of both human and mouse SASH1-Sam1 is practically identical. AF2 models of SASH1-Sam1 natural and cancer-associated variants can be overlayed on both backbone and heavy atoms, generating low RMSD values (Table S4 and Figure 5b–f). The greatest but still small variations can be observed in between the human native SASH1-Sam1 domain and its Y659C and L667P variants, especially within the ML region (Table S4 and Figure 5b,d). Interestingly, Y659 corresponds to Y652 in the SASH1 mouse primary structure (Figure 1), and we previously mentioned that this residue is important to mediate interactions with EphA8-Sam. Nevertheless, the Y652A mutation in mouse SASH1-Sam1 has been reported to abolish the binding to EphA8-Sam [9]. That said, our results likely point out that the Y659C substitution by affecting a conserved and rather buried residue (Table S1) might likely perturb the Sam-domain fold and consequently interfere with molecular recognition processes in which it is involved. It is also known that the introduction of a cysteine residue inside a protein is generally accompanied by destabilizing effects due to its tendency to form intermolecular disulfide bonds and favor aggregation phenomena.

As we have already pointed out in our previous study on cancer-related mutations affecting the EphA2-Sam/Ship2-Sam complex [58], much care needs to be taken when studying missense protein variants through AF2. In fact, AF2 is unable to very accurately predict the effects that missense mutations might induce in protein structures [59,60]. In fact, big structure perturbations or unfolding processes cannot be revealed by AF2, which will more likely predict protein variants carrying missense mutations and 3D models biased towards the wild-type structure [29]. In this context, the above-described comparison of AF2 models provides just some preliminary insights that will be better verified through many other computational investigations (see the next paragraphs).

The following steps of our protocol consist of predicting stability changes, induced by cancer-related mutations, in the diverse protein variants by focusing mostly on those affecting the ML binding site of SASH1-Sam1 (Figure 4). We used several bioinformatics/computational tools to evaluate changes in the Gibbs free energy (ΔΔG) caused by mutations. To get a more accurate portrait of stabilizing and destabilizing outcomes, it is important to compare and check the consistency in results obtained through different (ΔΔG) predictors [61,62,63]. We thus evaluated ΔΔG values through PopMuSiC (https://soft.dezyme.com/query/create/pop, access date 26 May 2024) [64], Maestro (Multi Agent Stability Prediction upon point mutations) (https://pbwww.services.came.sbg.ac.at/maestro/web/, access date 26 May 2024) [65], INPS-3D (Impact of Non Synonymous Variations on Protein Stability-3D) (https://inpsmd.biocomp.unibo.it/inpsSuite/default/index3D, access date 23 May 2024) [66], and FoldX [67,68], which represent structure-based instruments. PopMuSiC [64], Maestro [65], and FoldX [67,68] calculate ΔΔGs as differences in the folding free energy between the mutant and native (wt) variants (i.e., ΔΔG = ΔGfmut-ΔGfwt) so that negative ΔΔG values are associated with stabilizing mutations. Instead, INPS-3D [66] associates negative ΔΔG values with destabilizing effects, so the scores generated by this predictor have been inverted to be consistent among outcomes obtained by all the implemented prediction tools (Table 2) [69].

Results from ΔΔG predictors cannot be likely reputed accurate when mutations are associated with ΔΔG values within the ± 0.5 Kcal/mol range [63]. Regarding ΔΔG estimates from FoldX, predicted values higher than 2 standard deviations of the FoldX error (i.e., 1.6 Kcal/mol) can be intended as very accurate (=99% confidence range); however, ΔΔGs absolute values bigger than 0.8 Kcal/mol are still acceptable (95% confidence interval) [71]. With this in mind, in order to select stabilizing and destabilizing mutations, we adopted the following criteria [58]: 1—ΔΔG values were judged unreliable if dropping inside the ±0.5 Kcal/mol range for all predictors excluding FoldX; 2—concerning FoldX, a ± 0.8 kcal/mol reliability threshold was established; 3—destabilizing mutations were related to ΔΔG > 1 Kcal/mole in at least ¾ predictors; 4—stabilizing mutations were linked to ΔΔG < −1 Kcal/mole in at least ¾ predictors (Table 2).

According to these criteria, the SASH1-Sam1 variants, which are predicted to be destabilizing of the protein fold, are as follows: Y659C (Figure 4, Figure 5b and Figure S2c), L667P (Figure 4, Figure 5d and Figure S2e), R684S, and V686G (Figure 4). Interestingly, Y659C and L667P affect the ML Interaction interface. As pointed out before, Y659 is a residue important for the interaction with Sam domains from Eph receptors [9], and the tyrosine to cysteine substitution is expected to decrease protein solubility because of possible aggregation. Regarding the L667P mutation (Table S1), the substitution of leucine to proline could likely induce steric overlaps that will destabilize protein folding; the high Van Der Waals clash penalty (i.e., 2.92 Kcal/mol) revealed by FoldX (Table 2) [67] further supports this hypothesis.

Moreover, L667 (Figure 4, Figure 5d, and Figure S2e) is positioned at the beginning of the α3 helix, and the introduction of the helix-breaker proline residue within that position might perturb the SASH1-Sam1 structure by interfering with the hydrogen-bond network. Regarding other SASH1-Sam1 variants carrying mutations inside the ML binding site (i.e., D661H, E670D, D674N), the majority of ΔΔG predictions appear small and unreliable for most of them (Table 2), and this outcome, coupled with the good solvent exposure of D661 and E670 (around 30%) and D674 (around 21%) (Table S1), indicates that such mutations should not drastically affect protein folding but rather perturb the SASH1-Sam1-mediated protein–protein interaction network [72]. This is a plausible speculation considering that electrostatic interactions are important to stabilize Sam–Sam complexes mediated by SASH1-Sam1 [9], and the above-mentioned mutations involve all substitutions of negatively charged residues by either abolishing their charged state or reverting it (D661H) (Figure 4).

In addition, changes in melting temperatures induced by the mutations were evaluated with HotMusic (https://soft.dezyme.com/query/create/hot, access date 26 May 2024) [73,74] (Table S5). All cancer-related mutations, except R944Q, for which a very small change in Tm can be seen (Table S5), are predicted to cause a decrease in SASH1-Sam1 thermal stability, thus exerting a destabilizing effect [73,74]. However, the R944Q is a mutation positioned outside the ML interface (Figure 4).

To better understand a possible correlation between cancer-linked mutations and perturbation of the SASH1-Sam1 structure or interaction network, molecular dynamics simulations were conducted for those protein variants for which ΔΔG predictors failed to provide unambiguous results (i.e., D661H, E670D, and D674N) and could more likely negatively affect the formation of the Sam–Sam complex between SASH1-Sam1 and EphA2-Sam.

### 2.3. Molecular Dynamics

**Figure 6:**
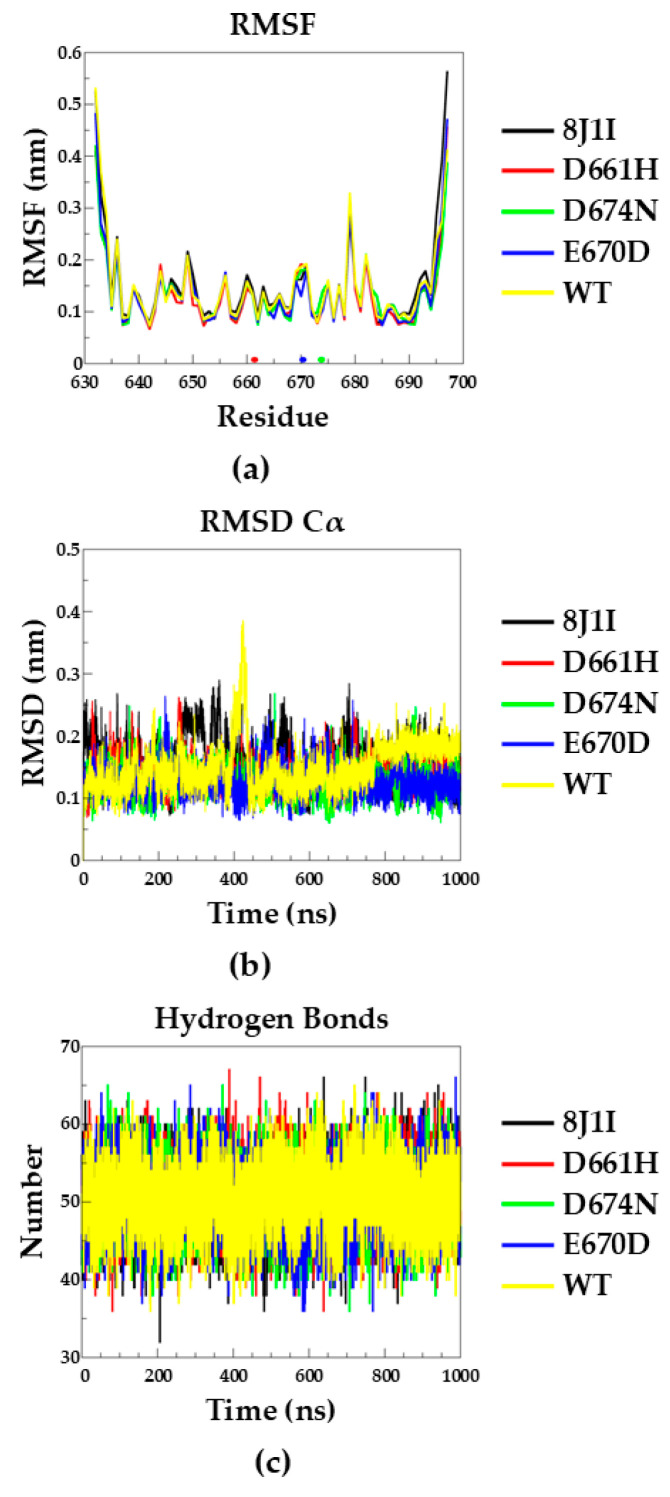
Main output parameters from molecular dynamics. (**a**) Residue RMSF profiles along the MD simulations of the SASH1 mutants. The amino acid residues of the 8J1I system were renumbered to be comparable to the others. (**b**) Residue RMSD profiles along the MD simulations, calculated considering the Cα atoms of the mutants and native SASH1-Sam1 variants. WT indicates that the AF2 model of SASH1-Sam1 was employed as the starting structure in the simulation; 8J1I indicates that the coordinates of the SASH1-Sam1 domain, extracted from the pdb X-ray structure 8J1I [9] of the SASH1-Sam1/EphA8-Sam complex, were used as inputs for MD studies. (**c**) Number of hydrogen bond interactions within each system computed along the MD simulations.

The impact of D661H, D674N, and E670D mutations on the structure and dynamics of SASH1-Sam1 was investigated using molecular dynamics simulations in explicit water. The trajectory analysis reveals that the residue fluctuations are quite comparable among the systems along the simulations, with only a marginal difference shown by the region 665–674 of the E670D variant, which presents a slightly lower flexibility if compared to the same region of the other systems (Figure 6a). The Cα atoms root mean squared deviations (RMSDs) analyses confirm that all the systems keep the starting arrangements without important deviations (Figure 6b). The wild-type human (indicated as “WT”) and mouse (indicated as “8J1I”) proteins are those showing the highest RMSD values with respect to the others (Figure 6b and Table S6); however, the global folds are maintained as confirmed by the high percentage of occurrence of hydrogen bonds, which are well preserved along the trajectories of all the systems (Figure 6c and Table S6). Representative frames extracted from the simulations based on RMSD criteria are shown in Figure S3 for all the SASH1-Sam1 proteins. It is evident from our calculations that the mutations considered are not affecting the protein structural organization, but the mutations are exposed to the solvent and probably impact the recognition and the binding to the partners.

Thus, these selected ML-related SASH1-Sam1 cancer-related variants and the native form were investigated by docking techniques against EphA2-Sam (see the following paragraphs).

### 2.4. Effect of Point Mutations on the Structure and Affinity of the EphA2-Sam/SASH1-Sam1 Complex

As mentioned in the Introduction section, structural information is available only for the mouse EphA8-Sam/SASH1-Sam1 complex, for which a crystal structure is deposited in the Protein Data Bank (PDB) [9]. As we are interested in studying the interaction between EphA2-Sam and SASH1-Sam1 and predicting how cancer-related mutations, which are positioned inside the SASH1-Sam1 ML interface, could affect the formation of this Sam–Sam association, we first had to generate a starting model of the EphA2-Sam/SASH1-Sam1 complex.

To achieve our goal, we implemented again AF2 with its “multimer routine” [53,75,76] using as input the primary sequences of human EphA2-Sam and SASH1-Sam1 (see Material and Methods section for further details). Before finalizing this step, we needed to establish a solid protocol to generate accurate predictions. To reach this goal, as a reference, we used the mouse EphA8-Sam/SASH1-Sam1 complex for which we generated an AF2 model and compared it with the experimental X-ray structure (pdb entry 8J1I [9]). Nevertheless, AF2 performance was also tested by exploiting the EphA4-Sam/SASH1-Sam1 system, as it is known that EphA4-Sam and SASH1-Sam1 are unable to bind to each other [9].

Next, as already reported in our previous publication on EphA2-Sam/Ship2-Sam cancer-associated mutations [58], we used the Haddock refinement interface and the AF2-generated models [28] to evaluate possible structural changes in the interaction topology and modulation of the binding strength induced by selected mutations. The PRODIGY web server was also tested to predict dissociation constant values [77], whereas LigPlot+ [11,31] was implemented to deeply analyze and compare patterns of intermolecular contacts among diverse human EphA2-Sam/SASH1-Sam1 variants.

#### 2.4.1. Generation of AF2 Starting Models of SASH1-Sam1 in Complex with Different Eph Receptors

First, the AF2 model of mouse EphA8-Sam in complex with SASH1-Sam1 was predicted by performing two different runs, one with the custom template option [78], using as a template for 3D model generation the crystal structure of the mouse EphA8-Sam/SASH1-Sam1 complex (pdb entry 8J1I [9]) (Figure S4 left panels) and another one by allowing ColabFold to look for optimal templates inside the PDB100 dataset (Figure S5 left panels) [79,80]; five AF2 models were predicted in each run. A set of AF2 scores was evaluated by us to judge the precision of the structure predictions. For our studies, we particularly considered the predicted Local Distance Difference Test (pLDDT) [7], the predicted Template Modeling (pTM), and interface pTM (ipTM) [76,81]. pLDDT indicates a per-residue evaluation of local confidence, and its value falls in the range of 0–100, where 100 stands for the highest accuracy; a pLDTT value equal to 80 or larger points out a rather good prediction [82,83]. The global accuracy of a whole protein–protein complex or just its interface can be related to the pTM and ipTM scores, respectively [75]. A pTM score lower than 0.5 likely indicates an inaccurate prediction. The accuracy of the predicted mutual orientations between the two proteins forming the Sam–Sam complexes can be linked instead to the ipTM scores, and in this case, good predictions are represented by ipTM values larger than 0.8, while values around 0.6 or lower likely point out inexact predictions, and finally, ambiguous predictions can be related to the 0.6–0.8 range of ipTM values [75].

Analysis of the AF2 prediction obtained with the custom 8J1I template for the EphA8-Sam/SASH1-Sam1 complex indicated that the best 4 predicted models had good accuracy scores and a binding topology resembling the ML/EH model (Figure S4). This observation was partially supported by RMSD evaluation, following superposition on the backbone atoms of each predicted model with the experimental X-ray structure (Figure S4a–d right panels and Table S7), which, however, highlighted some structure deviation from a canonical ML/EH topology in models 1 and 2 (Figure S4a,b right panels). In addition, the 5th predicted AF2 model (Figure S4e right panel) likely represents an inaccurate prediction characterized by a low ipTM value and not respecting the ML/EH binding topology (Figure S4e). Regarding the AF2 predictions obtained by searching a template within the PDB100 database (Figure S5), all five models resemble the ML/EH binding topology; the AF2 scores also indicated rather good predictions (Figure S5 left panels). Moreover, considering globally all five predicted models, the RMSD values highlighted a better agreement as well between experimental and predicted structures (Figure S5 right panels and Table S7). These results pointed out that by searching for the best template within the PDB100 database, more reliable structure predictions could be generated.

Similar AF2 runs and analyses were performed for the mouse EphA4-Sam/SASH1-Sam1 system (Figures S6 and S7 and Table S7), for which experimental data showed the absence of binding [9]. In this case, poor AF2 predictions, in terms of pTM and ipTM accuracy scores, were obtained for most of the models independently from the implemented choice of the template structure (Figures S6 left panels and S7 left panels). In addition, most predicted models did not present the ML/EH interaction topology, and large RMSD values were retrieved when comparing the 3D coordinates of the EphA4-Sam/SASH1-Sam1 system with the experimental structure of the EphA8-Sam/SASH1-Sam1 complex (Figures S6 and S7 right panels and Table S7). These outcomes produced by AF2 for the EphA4-Sam/SASH1-Sam1 system are interesting and might likely highlight a connection between experimentally observed absence of binding and inaccuracy of AF2 model predictions.

With this in mind, we opted to generate an AF2 model of the human EphA2-Sam/SASH1-Sam1 complex by AF2 by letting the algorithm search the template within the PDB100 database (Figure S8 left panels). The resulting five best models appear all characterized by good pLDDT, pTM, and ipTM scores and all follow the ML/EH model closely resembling the experimental crystal structure of EphA8-Sam in complex with SASH1-Sam1 (Figure S8 right panels and Table S7).

#### 2.4.2. Validation of Haddock and PRODIGY Computational Protocol

Before starting docking studies through the Refinement interface of Haddock [28] of the native human EphA2-Sam/SASH1-Sam1 complex and the selected cancer-related variants (i.e., EphA2-Sam/D661H SASH1-Sam1, EphA2-Sam/E670D SASH1-Sam1, and EphA2-Sam/D674N SASH1-Sam1), we had to validate our strategy. Thus, we checked if our in silico approach could succeed in producing results compatible with experimental available data; validation was also necessary to better comprehend how to analyze Haddock scores and PRODIGY outputs [58]. Again, as reported in the Introduction section, an experimental value of the dissociation constant for the binding of mouse EphA8-Sam with SASH1-Sam1 (K_D_ = 0.88 µM) is available as well as for the weaker interaction between EphA8-Sam and the Y652A SASH1-Sam1 mutant (K_D_ = ~885 µM) [9]. Therefore, we used the AF2-predicted models of the EphA8-Sam/SASH1-Sam1 complex as inputs in the Haddock Refinement Interface; a simple editing of the same pdb files with a text editor (see Material and Methods section) provided the starting models for the EphA8-Sam/Y652A SASH1-Sam1 complex [58,84]. We did not employ the experimental crystal structure of the EphA8-Sam/SASH1-Sam1 complex for protocol validation, as this crucial step had to simulate the exact in silico strategy we wanted to implement for the human EphA2-Sam/SASH1-Sam1 variants, for which experimental structure information is missing. The Haddock Refinement Interface performs refinement in water of protein complexes to optimize interface geometry and energetic parameters [28]. Overall, 100 output structures were obtained from each of the Haddock Refinement Interface runs performed for EphA8-Sam/SASH1-Sam1 and its EphA8-Sam/Y652A SASH1-Sam1 variant (Tables S8 and S9). In the case of the native EphA8-Sam/SASH1-Sam1 complex, 90 structures could be grouped into 6 clusters of conformationally related poses; for the EphA8-Sam/Y652A SASH1-Sam1 complex, 90 structures could be grouped into four clusters; in both cases, the best cluster in terms of Haddock scores also represented the most populated one (Table S8). We next evaluated the average Haddock scores, and PRODIGY predicted K_D_ (=dissociation constant) values considering the best 10 output models from the best and most populated clusters as well as those referring just to the best structure (i.e., the one with the lowest Haddock score within the specified clusters) (Table S9). A comparison of data indicated that for the native EphA8-Sam/SASH1-Sam1 complex, the PRODIGY predicted K_D_ value was larger, although not too discordant from the experimental one (Table S9); for the complex with the SASH1-Sam1 Y652A mutation, PRODIGY failed to predict the decrease in binding affinity that can be observed experimentally [9] (Table S9). However, and as already pointed out in our previous study on the cancer-related mutations affecting the EphA2-Sam/Ship2-Sam system [58], Haddock scores [28] reflect much better than PRODIGY-predicted K_Ds_ [77] changes in the interaction strength between two Sam domains. Indeed, for the EphA8-Sam/Y652A SASH1-Sam1 complex, an increase in the Haddock score values with respect to the native structure highlighted possibly a poorer interaction (Table S9).

In summary, this validation step showed that our strategy is rather useful to predict a perturbation of the binding in between two proteins induced by a point mutation through a comparison of Haddock scores [28] of native complexes with those referring to the cancer variants, while PRODIGY predicted dissociation constants [77] are less informative.

Next, we conducted similar computational studies of the EphA2-Sam/SASH1-Sam1 wild-type and cancer-related variants to assess how the selected missense mutations could affect binding between the two Sam domains.

#### 2.4.3. EphA2-Sam/SASH1-Sam1 Interaction

**Figure 7:**
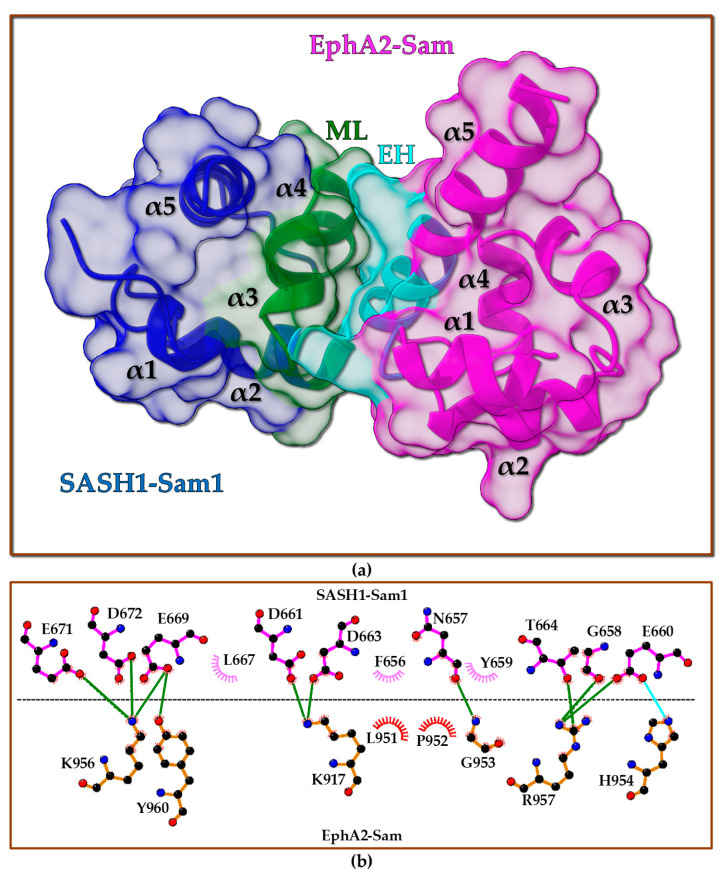
Best pose of the best and most populated Haddock [28] cluster of the EphA2-Sam/SASH1-Sam1 wild-type (WT) complex. (**a**) The blue and green colors indicate SASH1-Sam1 and its ML region (residues F656-L676, sequence numbering according to UniprotKB [30] entry O94885 for human SASH1), respectively. The magenta and cyan colors highlight EphA2-Sam and its EH region (residues I916-M918 and P952-Y960, sequence numbering according to UniprotKB [30] entry P29317 for human EphA2), respectively. (**b**) LigPlot+ [11,31] analysis of the Sam–Sam interaction interface: black, blue, and red spheres refer to carbon, nitrogen, and oxygen atoms, respectively; green and cyan solid lines refer to H-bonds and salt bridges, respectively; non-bonded interactions are represented by the crescents with bristles.

The five AF2 models of the human EphA2-Sam/SASH1-Sam1 complex were used as input structures in the Haddock Refinement Interface (Tables S10 and S11) [28]. In total, 87 out of 100 refined output models could be collected into 3 clusters (Table S10). The best cluster also represented the most populated one, and the best structure (in terms of Haddock score) within this cluster (Figure 7a) was further analyzed with LigPlot+ to detect intermolecular contacts at the Sam–Sam interface (Figure 7b) [11,31]. As mentioned before, the EphA2-Sam/SASH1-Sam1 complex has an ML/EH structural topology resembling the experimental structures of the EphA2-Sam/Ship2-Sam [21] as well as the EphA8-Sam/SASH1-Sam1 [9] complexes. A cluster of basic residues within the EH interface of EphA2-Sam (Figure 7b), including K956, K917, and R957, which have been reported to be crucial for stabilizing the EphA2-Sam/Ship2-Sam hetero-dimer [20,21], provide H-bond interactions with negatively charged residues on the ML interface of SASH1-Sam1 that are also involved in binding to EphA8-Sam [9] (Figure 1 and Figure 7b). As pointed out in the Introduction section (see also Figure 1), residue D656 in mouse SASH1-Sam1 is a crucial provider of intermolecular contacts with EphA8-Sam; in human SASH1-Sam1, this residue corresponds to D663 and is engaged in an intermolecular interaction with K917 from EphA2-Sam (Figure 7b). In the mouse EphA8-Sam/SASH1-Sam1 crystal structure, the residue Y652 from SASH1-Sam1 is another key contributor to intermolecular interactions [9]. In fact, Y652 is in close contact with the G978 residue, which is positioned at the beginning of the α5 helix in EphA8-Sam (Figure 1) and works as a sort of anchoring point to engage the ML interface of SASH1-Sam1 through a crucial backbone–backbone intermolecular H-bond with N650 (Figure 1). In our refined model of the EphA2-Sam/SASH1-Sam1 complex shown in Figure 7a, the same pattern of interactions can be observed as follows: Y659 (corresponding to Y652 in SASH1-Sam1) is facing a glycine residue positioned at the N-terminus of the α5 helix in EphA2-Sam (i.e., G953), which in turn provides the canonical H-bond with N657 (corresponding to N650 in mouse SASH1-Sam1), representing a sort of “signature” of ML/EH Sam–Sam complexes [12,58]. In fact, even in the EphA2-Sam/Ship2-Sam complex, G953 is involved in H-bonding with N1220 from the Ship2-Sam ML interface [20,21,58].

Additionally, other negatively charged residues that, according to mutagenesis studies [9], are crucially contributing to stabilizing the EphA8-Sam/SASH1-Sam1 complex (Figure 1), like E653, E662, and D665 (corresponding to residues E660, E669, and D672 from human SASH1-Sam1), are engaged in H-bonds or salt bridges with positively charged residues within the EH site of EphA2-Sam in the best model of the EphA2-Sam/SASH1-Sam1 complex (Figure 7b).

Therefore, our analysis indicates indeed that a very similar pattern of intermolecular interactions could characterize EphA2-Sam/SASH1-Sam1 (Figure 7), EphA2-Sam/Ship2-Sam, and EphA8-Sam/SASH1-Sam1 heterotypic associations [20,21,58].

#### 2.4.4. EphA2-Sam/D661H SASH1-Sam1 Interaction

**Figure 8:**
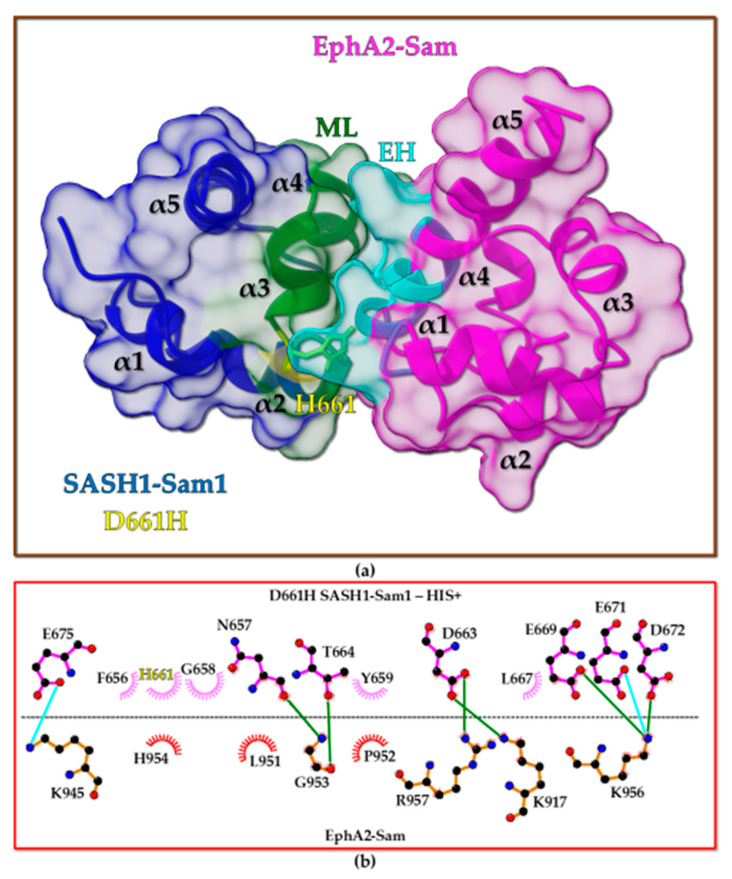
Best pose of the best Haddock [28] cluster of the EphA2-Sam/D661H SASH1-Sam1 complex. (**a**) The blue and green colors indicate D661H SASH1-Sam1 and its ML region (residues F656-L676), respectively. The side chain of H661 (“HIS+” protonation state) is shown in dark goldenrod. The magenta and cyan colors highlight EphA2-Sam and its EH region (residues I916-M918 and P952-Y960), respectively. (**b**) LigPlot+ [11,31] analysis of the interaction interface: black, blue, and red spheres refer to carbon, nitrogen, and oxygen atoms, respectively; green and cyan solid lines refer to H-bonds and salt bridges, respectively; non-bonded interactions are represented by the crescents with bristles.

Regarding the interaction between EphA2-Sam and D661H SASH1-Sam1, two Haddock runs were performed by keeping either H661 in the uncharged (Figure S9) or the positively charged states (Figure 8 and Figure S10).

Results from the Haddock Refinement interface indicate for the run performed considering the uncharged H661 that 90 out of 100 output models can be grouped into 4 clusters where the best cluster is also the most populated one (Table S10); for the run with the charged H661 state, 89 structures can be collected into five clusters, and in this case, the best cluster in terms of Haddock score does not correspond to the most populated one (Table S10). Consequently, we analyzed through LigPlot+ [11,31] the pattern of intermolecular contacts in the best EphA2-Sam/D661H SASH1-Sam1 output model from the best (=most populated) cluster carrying the uncharged mutated residues (Figure S9a) and both the best structures from the best (Figure 8a) and the most populated (Figure S10a) clusters deriving from the refinement conducted considering the charged histidine state (Table S10). The diverse EphA2-Sam/D661H SASH1-Sam1 refined complexes are still characterized by several interactions between negatively charged residues in the ML interface of D661H SASH1-Sam1 and positively charged residues from the EH site in EphA2-Sam (Figure 8b, Figures S9b and S10b). When H661 is in uncharged state (Figure S9b), it can be still engaged into intermolecular contacts with R957 from EphA2-Sam as no repulsions in between the positive charges of H and R will occur (Figure S9b); the pattern of electrostatic contacts appears partially diverse (Figure S9b) with respect to that in the native EphA2-Sam/SASH1-Sam1 complex (Figure 7b), although the crucial D663 residue (corresponding to D656 in mouse SASH1-Sam1) results similarly involved into H-bonding with K917, that is instead a key residue of the EH interface in EphA2-Sam (Figure S9b); the characteristic H-bond between G953 (EphA2-Sam) and N657 (corresponding to N650 in mouse SASH1-Sam1) and the contact with Y659 (corresponding to Y652 in mouse SASH1-Sam1) are also still preserved (Figure 7b and Figure S9b). When H661 is positively charged, in our model from the best cluster, it faces H954, and, under pH conditions that favor charged histidine states, a destabilizing effect is expected due to this interaction (Figure 8b); moreover, a lower total number of intermolecular H-bonds and salt bridges can be revealed with respect to the model of the native complex (Figure 7b), but the H-bond between G953 and N657 is still occurring (Figure 7b and Figure 8b).

In the best EphA2-Sam/D661H SASH1-Sam1 model from the most populated cluster carrying the charged mutated histidine (Figure S10), H661 is not involved in major intermolecular contacts, and also in this case, G953 and K917 (EphA2-Sam) are involved in key interactions with N657 and D663 (D661H SASH1-Sam1), respectively (Figure S10b), also characterizing the native complex (Figure 7b).

Analysis of Haddock scores (Table S11) shows that the mutation D661H causes an increase (i.e., less negative values) of the Haddock scores independently from the histidine protonation state that, as shown during protocol validation, should indicate a destabilizing effect of the Sam–Sam interaction with a possible reduction in binding strength. This is not surprising as electrostatic interactions between the positively charged EH site of EphA2-Sam and the negatively charged ML interface of SASH1-Sam1 are pivotal for Sam–Sam associations, and replacing within the ML region a negatively charged residue (i.e., D661), that in the model of the native complex (Figure 7) is involved in intermolecular contact with K917 from EphA2-Sam, with a histidine, that at certain pH values can be in the protonated state, is expected to induce electrostatic repulsions perturbing the binding in between the two Sam domains. The fact that the canonical H bond involving G953 (EphA2-Sam) at the N-terminus of the α5 helix and the interactions involving D663 are still preserved in the EphA2-Sam/D661H SASH1-Sam1 complex (Figure 8b, Figures S9b and S10b) might indicate that, in agreement with the increase in the Haddock scores with respect to the native model (Table S11), the D661H mutation in SASH1-Sam1 should just decrease the binding affinity for EphA2-Sam without totally hampering complex formation.

These in silico data let us speculate that the connection of the D661H SASH1-Sam1 mutation with cancer could be related to decreased SASH1-Sam1 binding to EphA2-Sam and that, being SASH1 associated with several anticancer activities (see the Introduction section), SASH1-Sam1 could engage EphA2-Sam to inhibit its pro-oncogenic signaling.

#### 2.4.5. EphA2-Sam/E670D SASH1-Sam1 Interaction

Concerning the EphA2-Sam/E670D SASH1-Sam1 complex (Figure S11a), the clusterization procedure of the 100 refined models generated by the Haddock Refinement Interface [28] (Table S10) grouped 94 output structures into 3 conformational families (=clusters) where the best cluster also corresponded to the most populated one. The intermolecular contacts at the Sam–Sam interface were analyzed with LigPlot+ [11,31] in the best model of the best (=most populated) cluster (Figure S11b). E670 belongs in SASH1-Sam1 (Figure 2) to a cluster of negatively charged residues 669-EEED-672; in the best model from the best cluster of the native EphA2-Sam/SASH1-Sam1 complex (Figure 7a), E670 is not engaged in intermolecular contacts at the Sam–Sam binding interface, while several interactions are provided by E671, D672, and E669 (Figure 7b). Similarly, in the EphA2-Sam/E670D SASH1-Sam1 model (Figure S11a), E669 and D672 are contributing intermolecular contacts (Figure S11b); in both the best models of native EphA2-Sam/SASH1-Sam1 and its E670D variant, the crucial H-bond by G953 (EphA2-Sam) and the possible crucial pattern of interactions provided by D663 are maintained (Figure 7b and S11b). Inspection of Haddock scores (Table S11) further points out that this mutation should not significantly alter the overall structural organization and the binding affinity of EphA2-Sam towards SASH1-Sam1, possibly showing that its relationship to cancer might be due to different signaling pathways not involving EphA2 but other SASH1-Sam1 binding partners. This outcome is plausible considering that the E670D mutation in SASH1 is replacing a negatively charged residue with another acidic residue within a short amino acid sequence rich in this kind of amino acids, which can mutually switch their role in providing electrostatic interactions with the positively charged EH site of EphA2-Sam.

#### 2.4.6. EphA2-Sam/D674N SASH1-Sam1 Interaction

Out of 100 refined Haddock models of the EphA2-Sam/D674N SASH1-Sam1 complex, 93 could be grouped into two clusters (Table S10). We analyzed the best output structure from the best and most populated cluster (Figure S12a). In the best model of the native EphA2-Sam/SASH1-Sam1 complex, D674 is not engaged in interactions with EphA2-Sam (Figure 7b); in the best-analyzed model of EphA2-Sam in complex with the D674N SASH1-Sam1 variant, N674 is still not providing intermolecular contacts (Figure S12b). Similarly to the native complex (Figure 7b), the EphA2-Sam/D674N SASH1-Sam1 complex (Figure S12a) appears mainly stabilized by electrostatic interactions between residues from the ML and EH interfaces; G953 (EphA2-Sam) is engaged in H-bonding with N657 (D674N SASH1-Sam1) and is also contacting Y659 (D674N SASH1-Sam1); D663 makes contacts with K917 (EphA2-Sam). Interestingly, as previously pointed out, this interaction network closely resembles that observed in the mouse EphA8-Sam/SASH1-Sam1 crystal structure (Figure 1). The Haddock scores related to the wild-type EphA2-Sam/SASH1-Sam1 models and those related to the EphA2-Sam/D674N SASH1-Sam1 variant (Table S11) are not very different from each other and possibly indicate, along with LigPlot+ analyses (Figure 7b and Figure S12b), a rather similar structural topology of binding and interaction affinity (Table S11). This analysis, similarly to what was revealed for the E670D mutation, likely points out that the correlation between the D674N SASH1-Sam1 variant and cancer might not be due to SASH1-Sam1 binding to EphA2 but involve a different interaction partner.

## 3. Materials and Methods

### 3.1. Sequence Alignment

Sequence alignments shown in Figure 2 and Figure 3 were generated with the Clustal Omega program for multiple sequence alignment [85] (https://www.ebi.ac.uk/Tools/msa/clustalo/, access dates 2 and 31 July 2024). The residues with negatively charged side chains (D and E) are indicated in blue. The residues with positively charged side chains (K and R) are indicated in red. The residues with polar side chains (N, Q, S, and T) are violet. The residues with aromatic side chains (H, F, W, and Y) are indicated in magenta. The residues with apolar aliphatic side chains (A, I, L, M, and P, V) and G are indicated in green; “*” represents identical residues among the diverse sequences, “:” represents conservative substitutions, and “.” stands for semi-conservative substitutions.

Regarding SASH1 (Figure 2), the following sequences were overlayed: SASH1 from Homo sapiens (UniProtKB [30] code O94885, range P632-D698), SASH1 from Mus musculus (UniProtKB [30] code P59808, residues P625-D691), and the sequence from the X-ray structure (pdb entry 8J1I [9], chain H with the N-terminal residues GPGSEF deriving from the plasmid). Secondary structure elements for SASH1-Sam1 reported on top of Figure 2 (i.e., α1 V638-I645, α2 K648-L655, α3 L662-F665, α4 E670-E675, and α5 P681-E696) refer to UniprotKB [30] entry O94885 and were defined based on MolMol [86] inspection of the X-ray structure (pdb code 8J1I, chain H). The ML interfaces (Figure 2) include the following residues: F656-L676 (for Homo sapiens SASH1, UniProtKB [30] code O94885), F649-L669 (for Mus musculus SASH1-Sam1, UniProtKB [30] code P59808), and F649-L669 (for Mus musculus SASH1-Sam1 from the chain H of the X-ray structure pdb entry 8J1I [9]).

Regarding Eph receptors (Figure 3), the following sequences were aligned: Homo sapiens EphA2 (UniProtKB [30] code P29317, range V904-I976 and encompassing the Sam domain (residue range V904-Q968)), Mus musculus EphA2 (UniProtKB [30] entry Q03145, residues V905-I977, encompassing the Sam domain (residue range V905-Q969)), Mus musculus EphA8 (UniProtKB [30] entry O09127, residues L932-L1004, encompassing most of the Sam domain (N929-Q993)), and Mus musculus EphA4 (UniProtKB [30] code Q03137, residues V914-V986, covering most of the Sam domain (S911-Q975)).

Secondary structure elements for EphA2-Sam reported on top of Figure 3 (i.e., α1 V909-I916, α2 Q919-A928, α3 I933-V936, α4 N941-R946, and α5 P952-N970 refer to UniprotKB [30] entry P29317) were defined based on MolMol [86] inspection of the NMR structure (pdb code 2E8N, first conformer). The EH interfaces (Figure 3) are defined as follows: residues I916-M918 and P952-Y960 for Homo sapiens EphA2-Sam (UniProtKB [30] code P29317); residues I917-M919 and P953-Y961 for Mus musculus EphA2-Sam (UniProtKB [30] code Q03145); residues I941-M943 and M977-G985 for Mus musculus EphA8-Sam (UniProtKB [30] code O09127); and residues I923-M925 and I959-S967 for Mus musculus EphA4 (UniProtKB [30] code Q03137).

### 3.2. Editing of the SASH1-Sam1 Structure

The atomic coordinates of SASH1-Sam1 were extracted from the X-ray structure of the SASH1-Sam1/EphA8-Sam complex (pdb entry code 8J1I [9]) by following a multistep protocol: (1) water molecules were removed; (2) the coordinates of EphA8-Sam (i.e., chain 8) were removed so as to keep just chain H corresponding to SASH1-Sam1; (3) the F624 residue not belonging to the Mus musculus SASH1 sequence (UniprotKB [30] entry P59808), but deriving from the protein construct, was removed from the pdb file; (4) the missed side chains (K641, K659, E689, and Y690) or side chain with multiple conformations (D656) were reconstructed by the macro “ReconstructSideChains” of FoldX 5 [67]. Next, the coordinates of SASH1-Sam1, isolated from the X-ray structure of the complex with EphA8-Sam, were optimized by the macro “RepairPDB” of FoldX 5 [67], which fixed bad torsion angles and/or Van der Waals’ clashes and optimized side chains’ rotameric states to gain the lowest energy configuration [67]. Three optimization cycles were repeated to obtain neglecting energy difference values, below the FoldX accuracy range (i.e., 0.8 Kcal/mol), between structures referring to two consecutive optimization runs [71]. Next, the non-polar hydrogens were added with UCSF Chimera [70].

### 3.3. AlphaFold2 Models

#### 3.3.1. SASH1-Sam1

AF2 [53,54], executed through the ColabFold server [87,88] (https://colab.research.google.com/github/sokrypton/ColabFold/blob/main/AlphaFold2.ipynb#scrollTo=kOblAo-xetgx, access date 23 May 2024), was employed to predict models of the wild-type SASH1-Sam1 domain (residues P632-Y697 from UniProtKB [30] entry O94885 for human SASH1) and cancer-related missense mutants (Y659C, D661H, L667P, E670D, and D674N). AF2 predictions were run using the X-ray structure of the SASH1-Sam1/EphA8-Sam complex (pdb entry code 8J1I [9]) as a custom template model. For each protein variant, five structures were predicted, and all of them were selected for post-prediction relaxation via gradient descent in the Amber force field. Default settings were used for all options except the number of seeds, which was set equal to 1. Generated AF2 models were next optimized by the macro “RepairPDB” of FoldX 5 [71] (3 cycles), and non-polar hydrogens were added with UCSF Chimera [70].

#### 3.3.2. SASH1-Sam1 in Complex with EphA4, EphA8, and EphA2-Sam WT

AF2 [53,54] was exploited to get the models of SASH1-Sam1 in complex with different Eph receptors. About computational method validation, the amino acid sequence of Mus musculus SASH1-Sam1 (UniProtKB [30] entry P59808, residues P625-D691) and its Y652A variant, as well as those from Mus musculus EphA8-Sam (UniProtKB [30] entry O09127, residues L932–L1004), along with Mus musculus EphA4-Sam (UniProtKB [30] entry Q03137, residues V914–V986), were considered as inputs to predict models of Sam–Sam complexes [9]. The structures were obtained through two different runs, one by setting “pdb100” as the “template_mode” option and the other by setting “custom” as the template_mode option and choosing the pdb structure 8J1I [9] (both chains) as the custom template. In all cases, “mmseqs2_uniref_env”, “unpaired_paired”, and “alphafold2_multimer_v3” were chosen as settings for “msa_mode” (MultiSe-quenceAlignment), “pair_mode”, and “model_type”, respectively. The “1”, “512:1024”, “48”, and “0.0” values were selected for “num_seeds”, “max_msa”, “num_recycles”, and “recycle_early_stop_tolerance” parameters, respectively. For each protein complex, five structures were predicted, and post-prediction relaxation via gradient descent in the Amber force field was achieved for each of them. In the end, non-polar hydrogens were added to each model with UCSF Chimera [70]. The structure of the Homo sapiens wild-type SASH1-Sam1 domain (residues P632-Y697 from UniProtKB [30] entry O94885) in complex with the Homo sapiens EphA2-Sam domain (residues T908-V972 from UniProtKB [30] entry P29317) was similarly predicted by AF2 [53] by employing the same settings of the runs associated with the Mus musculus Sam–Sam complexes described before. The only exception was the choice to run only the prediction based on the “pdb100” option of the “template_mode” parameter. The pLDDT (predicted Local Distance Difference Test) [7], predicted Template Modeling (pTM), and interface pTM (ipTM) values [75,76,81,83] were analyzed to evaluate the structural prediction accuracy for each complex.

### 3.4. Bioinformatic Analyses. Structure-Based Predictions

#### 3.4.1. Analysis of Conserved Residues

The ConSurf web server was employed to evaluate the evolutionary conservation for the amino acids of SASH1-Sam1 affected by the cancer-associated mutations collected in the COSMIC catalogue [41] (http://consurf.tau.ac.il, access date 24 May 2024). The predictions were run with default parameters using as input the AF2 predicted structure (best model) of human SASH1-Sam1 WT after the optimization steps described above.

#### 3.4.2. Thermodynamic Stabilities (ΔΔG Evaluation)

Gibbs free energy changes (i.e., ΔΔG = ΔG Mutant − ΔG Wild-Type) were estimated by the following different online tools: PoPMuSiC [64] (Prediction of Protein Mutant Stability Changes) (https://soft.dezyme.com/, access date 26 May 2024), INPS-3D [66] (Impact of Non-synonymous mutations on Protein Stability) (http://inpsmd.biocomp.unibo.it, access date 23 May 2024), and MAESTRO (Multi AgEnt STabil-ity pRedictiOn) [65] (http://biwww.che.sbg.ac.at/MAESTRO, access date 26 May 2024); MAESTRO predictions refer to pH 7. In addition, the BuildModel macro from FoldX 5 [67,71,89,90] was also used for ΔΔG evaluation. The input structure was the AF2 human SASH1-Sam1 WT best model after undergoing the optimization steps described in Section 3.3.1.

#### 3.4.3. Thermal Stability (ΔTm Evaluation)

ΔTm values (i.e., variations in melting temperatures calculated as differences Tm Mutant-Tm Wild-Type) were estimated with the HoTMuSiC web server [73] (https://soft.dezyme.com/, access date 26 May 2024). The predictions were launched starting from the AF2 best model of SASH1-Sam1 without employing Tm experimental values.

### 3.5. Molecular Dynamics

Molecular dynamic simulations were run for different human SASH1-Sam1 variants (i.e., the wild-type (indicated as “WT”) and cancer-related mutants) modeled with AF2 [53] along with the native Mus musculus SASH1-Sam1 X-ray structure extracted from pdb entry 8J1I [9] (indicated as “8J1I”) for 1 µs using GROMACS 2024.2 [91]. Molecules were solvated with an octahedron box of TIP3P water model [92] and neutralized with Na^+^ and Cl^-^ counterions. MD simulations were run using PBC (Periodic Boundary Conditions), and constraints on H-bond lengths were set through the LINCS (Linear Constraint Solver) algorithm [93]. Other MD simulation parameters include the following: an integration time step of 2 fs, the particle mesh Ewald method to treat electrostatics, and a non-bonded cut-off for the Lennard-Jones potential [94]. In addition, the following algorithms, C-rescale and V-rescale [95], were employed to monitor pressure (atm = 1) and temperature (T = 300 K), respectively. Water molecules were relaxed by energy minimization and followed by 10 ps MD at 300 K, harmonically restraining the atomic positions. The temperature gradually increased (from 50 to 300 K) through a six-step route, followed by a short 5 ps equilibration stage at 300 K at NPT (constant particle number, constant pressure, and constant temperature). Each SASH1-Sam1 variant was run under NPT conditions without restraints for 1 µs. The trajectories were analyzed through the following diverse software: GROMACS-2024.2 [96], PyMOL GLSL version 4.60 [97], and VMD (Visual Molecular Dynamics) for LINUXAMD64 (version 1.9.4a37) [98]. The trajectory states were clustered, and the representative molecular dynamics conformations were selected as those exhibiting the lowest RMSD values relative to the other members of the most populated cluster.

### 3.6. Haddock Studies: The Refinement Interface

#### 3.6.1. Validation Protocol

The five AF2 [53] models of the complex between the Mus musculus SASH1-Sam1 domain (residues P625-D691 from UniProtKB [30] entry P59808) and the Mus musculus EphA8-Sam domain (residues L932-L1004 from UniProtKB [30] entry O09127) were used as inputs for the Refinement Interface of the Haddock web server (version 2.4) (https://wenmr.science.uu.nl/haddock2.4/refinement/, access date 10 September 2024) [28,99,100]. The protonation states found for histidine residues in the AF2 models [53] (i.e., “HISD” for H949, H979, and H1003 from EphA8-Sam and H676 from SASH1-Sam1; “HISE” for H643 from SASH1-Sam1) were preserved during the Haddock Refinement by setting them in the “his_patch” section of the “job_params.json” files. The Haddock web server provided 100 output structures for each analyzed complex, which were clustered by setting “RMSD” as the method, using 0.9 Å as the cutoff value, and the lowest cluster size equal to 4. The cutoff value was chosen after performing several Haddock runs and ensuring the highest number of clustered models. Next, for each complex, the best 10 structures of the best and/or most populated clusters were selected, and their average Haddock scores were calculated with the corresponding errors (expressed as population standard deviation). In addition, Haddock scores for just the best structures (i.e., the ones with the lowest Haddock scores) of either the best and/or most populated clusters were kept into account. The same Haddock protocol was applied to the Mus musculus Y652A SASH1-Sam1 mutant in complex with EphA8-Sam. The mutation was introduced in the native SASH1-Sam1/EphA8-Sam complex by editing the corresponding pdb file and replacing the three-letter amino acid code (TYR) with the substituted amino acid (ALA) before running the Haddock Refinement Interface analysis [28,84]. The PRODIGY web server (https://wenmr.science.uu.nl/prodigy/, access date 10 September 2024) [77] was employed to get the dissociation constant (K_D_) values for EphA8-Sam/SASH1-Sam1 native and non-native complexes starting from the output models generated by the Haddock Refinement Interface. The K_D_ values were acquired adopting the same criteria used for the Haddock scores (i.e., considering either the best structures or averaging the values over the best 10 structures in both the best and the most populated clusters).

#### 3.6.2. Generation and Analyses of EphA2–Sam/SASH1-Sam1 WT and Mutated Complexes

The five AF2 [53] models of the complex between the Homo sapiens SASH1-Sam1 domain (residues P632-Y697 from UniProtKB [30] entry O94885) and the Homo sapiens EphA2-Sam domain (residues T908-V972 from UniProtKB [30] entry P29317) were used as inputs for the Refinement Interface of the Haddock web server (version 2.4) (https://wenmr.science.uu.nl/haddock2.4/refinement/, access date 27 September 2024) [28,84]. The protonation states found for the histidine residues from EphA2-Sam (i.e., “HISD” for H924 and H954) and from SASH1-Sam1 (i.e., “HISE” for H650 and “HISD” for H683) were preserved during Haddock refinement runs. All studied SASH1-Sam1 mutations (i.e., D661H, E670D, and D674N) were introduced inside the pdb coordinates of the wild-type complex by replacing the native three-letter amino acid code with that corresponding to the substituted residue before running the Haddock Refinement Interface [28,84]. Concerning the complex with the D661H SASH1-Sam1 variant, two distinct runs were performed by using either the protonation state predicted by AF2 in the single D661H SASH1-Sam1 domain prediction (i.e., “HISD”) or the “HIS+” charged state. Histidine configurations were set in the “his_patch” section of the “job_params.json” files. The analysis of Haddock scores [28] was conducted by adopting the same criteria used for the protocol validation (paragraph 3.6.1).

Figures of diverse Sam–Sam complexes, including translucent surface representations, were produced with ChimeraX (version 1.5) [101].

### 3.7. Diagrams of Intermolecular Contacts by LigPlot+

2D diagrams representing the main intermolecular contacts characterizing each native and mutated Sam–Sam complex were obtained by the LigPlot+ (version 2.2.8) software [11,31]. To detect H-bonds, the default cut-off limits were considered (i.e., cut-offs for H-acceptor and donor-acceptor distances equal to 2.7 Å and 3.35 Å, respectively). All non-bonded contacts were identified by employing the default values as cut-offs for minimum and maximum distances (=2.9 Å and 3.9 Å, respectively). Salt bridges involving positively and negatively charged amino acids were retrieved by considering a 4.0 Å maximum mutual distance, as indicated in reference [102]. The structures analyzed with LigPlot+ consisted of the best output models of the best and/or most populated clusters obtained as outputs from the Haddock Refinement Interface [28].

## 4. Conclusions

We employed a computational strategy to analyze cancer-related mutations in the first Sam domain of the adaptor protein SASH1 from a structural point of view. SASH1 has been lately identified as a novel interactor of several Eph receptors (including EphA8 and EphA2), which are engaged through heterotypic interactions involving the Sam (Sterile alpha motif) domains [9]. The binding of EphA8-Sam to SASH1-Sam1 has been both functionally and structurally well-characterized [9], but little is known about the EphA2-Sam/SASH1-Sam1 association. Studying the EphA2-Sam/SASH1-Sam1 complex in the context of SASH1-Sam1 cancer-related variants could provide important insights for applications in the anticancer drug discovery field. In fact, SASH1 is a known tumor suppressor protein [1], whereas EphA2, although exhibiting often controversial functions, is associated with several pro-cancer activities [4]; thus, this Sam–Sam interaction could be important to modulate the EphA2 cancer-related signaling pathway. Nevertheless, it cannot be excluded that the EphA2-Sam/SASH1-Sam1 system could be employed as a target to discover novel anticancer therapeutics.

A similar in silico approach to that implemented here has already been recently exploited in our laboratory to study cancer-related mutations affecting the EphA2-Sam/Ship2-Sam complex [58], representing another protein–protein interaction mediated by EphA2-Sam that is mainly linked to pro-oncogenic outcomes [20].

Following our approach, we first searched the COSMIC [27] catalogue for cancer-related missense mutations in the SASH1-Sam1 domain. Next, we selected those amino acid substitutions positioned inside the so-called ML site, which is responsible for the interaction of SASH1-Sam1 with the EH interface of partner Sam domains. Then, 3D models of different cancer-related SASH1-Sam1 variants were built with AF2 [53]. Primary sequence and 3D structure analyses through a variety of bioinformatic tools were conducted, and the results were further checked by molecular dynamics simulations. This first stage was useful to identify SASH1-Sam1 mutated forms more likely associated with destabilization of the SASH1-Sam1 domain fold and select a few variants that could instead hamper the interaction between SASH1-Sam1 and binding partners. The latter mutations were more deeply analyzed in the context of the EphA2-Sam/SASH1-Sam1 complex by docking studies. As experimental structure information is not available for the interaction between human EphA2-Sam and SASH1-Sam1 but just for mouse EphA8-Sam in complex with SASH1-Sam1 [9], we generated a 3D model of the EphA2-Sam/SASH1-Sam1 complex with AF2. This model presents the ML/EH binding topology canonical of Sam–Sam interactions [5], where SASH1-Sam1 and EphA2-Sam form the ML and EH interfaces, respectively. Moreover, the model obtained for the EphA2-Sam/SASH1-Sam1 complex closely resembles the experimental structures of the EphA2-Sam/Ship2-Sam [20,21] and EphA8-Sam/SASH1-Sam1 [9] heterodimers, preserving a very similar pattern of intermolecular contacts as well. Further studies with the Haddock refinement interface [28] were next carried out to verify how a few selected cancer-related mutations could affect the binding of EphA2-Sam to SASH1-Sam1. Our computational study suggested that a few cancer-related SASH1-Sam1 variants, with mutations inside the ML interface (i.e., L667P and Y659C), could more likely disturb the Sam1 domain fold and consequently avoid interaction between EphA2-Sam and SASH1-Sam1 through a kind of indirect mechanism. However, it is worth noting that Y659C (corresponding to Y652 in the SASH1 mouse sequence) is crucial to mediate interactions with EphA8-Sam, and the mutation Y652A hampers binding to EphA8-Sam [9].

Instead, the D661H mutation could directly weaken the interaction between SASH1-Sam1 and EphA2-Sam without inducing much perturbation of the SASH1-Sam1 structure. Other SASH1-Sam1 variants, like the E670D and the D674N, are predicted to have a marginal effect on the stability of SASH1-Sam1 alone and on its interaction with EphA2-Sam, thus letting us speculate that their correlation to cancer could be associated with inhibition of SASH1-Sam1-mediated interactions with partners different from EphA2-Sam.

In the end, this preliminary computational study highlights that the relationship to cancer of certain SASH1-Sam1 mutations (like the Y659C and D661H) could be due to negative modulation of the SASH1-Sam1/EphA2-Sam association.

From a functional point of view, our results on the D661H SASH1-Sam1 variants provide insightful mechanistic insights, letting us speculate that the tumor suppressor protein SASH1 could be engaged at the EphA2 receptor site by means of the heterotypic Sam–Sam association to downregulate its pro-oncogenic signaling. Of course, experimental validation and in vitro assays in diverse cancer cell lines are needed to undoubtedly associate antioncogenic effects with the EphA2-Sam/SASH1-Sam1 complex.

## Figures and Tables

**Table 2:** Variations in SASH1-Sam1 stability due to cancer-linked mutations. ΔΔG estimates were obtained through the indicated bioinformatic tools. Mutations affecting residues inside the ML binding site (F656-L676) have been underlined. ΔΔG values possibly associated with more destabilizing mutations (i.e., >1 Kcal/mol) are highlighted in red.

SASH1-Sam1 ^@^	PopMuSiC ΔΔG (Kcal/mol)	Maestro ΔΔG (Kcal/mol) /c_pred_^#^	INPS-3D ΔΔG (Kcal/mol)	FoldX ΔΔG (Kcal/mol) /Δ(VdW) ^$^
P633L	0.11	−0.11/0.92	−0.33	0.75/−0.99
R644Q	0.12	−0.85/0.86	0.47	1.65/−0.04
Y659C	1.94	1.83 / 0.89	1.99	2.20/−0.03
D661H	0.38	0.63/0.90	0.46	2.94/−0.0003
L667P	1.17	0.76/0.86	1.81	2.83/2.92
E670D	0.30	0.31/0.95	0.39	1.14/−0.0002
D674N	0.14	0.31/0.91	0.27	0.84/0.73
R679S	0.31	−0.42/0.91	0.41	1.25/−0.00005
P681L	0.74	−0.35/0.83	0.38	1.47/−0.002
P681S	0.57	−0.35/0.88	0.96	1.86/0.01
R684S	1.95	1.63 / 0.90	1.53	3.18/0.38
V686G	1.47	1.32 / 0.84	2.59	1.18/−0.02
L687I	1.22	0.77/0.91	−0.27	1.53/1.37
L688F	1.28	0.98/0.87	1.26	1.12/−0.002

^@^ The AF2 model of SASH1-Sam1 (WT) (residue range P632-Y697) was employed as input for the analysis after 3 cycles of the FoldX RepairPDB macro [67] and the addition of protons by UCSF Chimera [70]. ^#^ Maestro predicted confidence (c_pred_) [65]. ^$^ Δ(VdW) clashes are the differences in Van der Waals clashes between models carrying and not carrying the specified mutations.

## Data Availability

Most data are included within the main text and supplementary material. However, further details will be provided by the corresponding author upon reasonable request.

## Supplementary Materials

The following Supporting Tables and Figures can be downloaded at: https://www.mdpi.com/article/10.3390/molecules30030718/s1. Table S1. COSMIC missense mutations. Table S2. Primary sequence analyses by EXPASY. Table S3. RMSD values for the comparison of 3D coordinates of human WT and mouse SASH1-Sam1 domains. Table S4. RMSD values for the comparison of 3D coordinates of human WT and mutated SASH1-Sam1 domains. Table S5. HoTMuSiC output. Table S6. RMSD values and hydrogen bonds along MD simulations. Table S7. RMSD evaluation: the X-ray structure of the EphA8-Sam/SASH1-Sam1 complex vs each of the best five AF2 models of mouse EphA8-Sam/SASH1-Sam1, mouse EphA4-Sam/SASH1-Sam1 and human EphA2-Sam/SASH1-Sam1. Tables S8 and S9. Validation of the in silico strategy (Haddock Refinement Interface and Prodigy). Table S10. Haddock refinement: number of clusters and relative populations. Table S11. Haddock scores. Figure S1. Sequence identity assessment. Figure S2. AF2 models of human SASH1-Sam1 variants; X-ray structure of mouse SASH1-Sam1 (8J1I). Figure S3. The representative models derived from MD simulations. Figure S4. The best five AF2 models of mouse EphA8-Sam/SASH1-Sam1 complex (template mode) and comparison with the EphA8-Sam/SASH1-Sam1 X-ray structure. Figure S5. The best five AF2 models of mouse EphA8-Sam/SASH1-Sam1 complex, generated with the PDB100 template mode and comparison with the EphA8-Sam/SASH1-Sam1 X-ray structure. Figure S6. The best five AF2 models of mouse EphA4-Sam/SASH1-Sam1 complex, generated by employing the pdb structure 8J1I as template and comparison with the EphA8 Sam/SASH1-Sam1 X-ray structure. Figure S7. The best five AF2 models of mouse EphA4-Sam/SASH1-Sam1 complex generated with the PDB100 template mode and comparison with the EphA8-Sam/SASH1-Sam1 X-ray structure. Figure S8. The best five AF2 models of human EphA2-Sam/SASH1-Sam1 complex, generated with the PDB100 template mode and comparison with the EphA8-Sam/SASH1-Sam1 X-ray structure. Figure S9. Best structure from the best and most populated Haddock cluster for the EphA2-Sam/D661H SASH1-Sam1 complex with H661 in the uncharged state, and summary of intermolecular interactions. Figure S10. Best structure from the most populated Haddock cluster for the EphA2-Sam/D661H SASH1-Sam1 complex with H661 in the positively charged state, and summary of intermolecular interactions. Figure S11. Best structure from the best and most populated Haddock cluster for the EphA2-Sam/E670D SASH1-Sam1 complex and summary of intermolecular interactions. Figure S12. Best structure from the best and most populated Haddock cluster for the EphA2-Sam/D674N SASH1-Sam1 complex and summary of intermolecular interactions.
