# Supplementary material for: Sam-Sam Association Between EphA2 and SASH1: In Silico Studies of Cancer-Linked Mutations
Source: Molecules. 2025 Feb 5;30(3):718. doi: 10.3390/molecules30030718 (PMC11820823; doi:10.3390/molecules30030718)
Supplement: Supplementary file 1 [file molecules-30-00718-s001.zip › Supplementary Figures.pdf]

Supplementary Figures

Figure S1. Sequence identity assessment: EphA2 (Homo sapiens) vs EphA2 (Mus musculus)/EphA8 (Mus musculus)/ EphA4 (Mus musculus) and EphA2 (Mus musculus) vs EphA8 (Mus musculus)/EphA4 (Mus musculus).
Figure S2. AF2 models of human SASH1-Sam1 wild-type (WT) and cancer-related mutants; X-ray structure of mouse SASH1-Sam1 (8J1I).
Figure S3. The representative models derived from MD simulations of diverse SASH1-Sam1 variants (human WT and cancer-related proteins, mouse X-ray structure 8J1I).
Figure S4. The best five AF2 models of mouse EphA8-Sam/SASH1-Sam1 complex, generated by employing the pdb structure 8J1I as template, with accuracy scores. Comparison with the EphA8-Sam/SASH1-Sam1 X-ray structure.
Figure S5. The best five AF2 models of mouse EphA8-Sam/SASH1-Sam1 complex, generated with the PDB100 template mode, with accuracy scores. Comparison with the EphA8-Sam/SASH1-Sam1 X-ray structure.
Figure S6. The best five AF2 models of mouse EphA4-Sam/SASH1-Sam1 complex, generated by employing the pdb structure 8J1I as template, with accuracy scores. Comparison with the EphA8-Sam/SASH1-Sam1 X-ray structure.
Figure S7. The best five AF2 models of mouse EphA4-Sam/SASH1-Sam1 complex generated with the PDB100 template mode, with accuracy scores. Comparison with the EphA8-Sam/SASH1-Sam1 X-ray structure.
Figure S8. The best five AF2 models of human EphA2-Sam/SASH1-Sam1 complex, generated with the PDB100 template mode, with accuracy scores. Comparison with the EphA8-Sam/SASH1-Sam1 X-ray structure.
Figure S9. Best structure from the best and most populated Haddock cluster for the EphA2-Sam/D661H SASH1-Sam1 complex with H661 in the uncharged state, and summary of intermolecular interactions by LigPlot+.
Figure S10. Best structure from the most populated Haddock cluster for the EphA2-Sam/D661H SASH1-Sam1 complex with H661 in the positively charged state, and summary of intermolecular interactions by LigPlot+.
Figure S11. Best structure from the best and most populated Haddock cluster for the EphA2-Sam/E670D SASH1-Sam1 complex and summary of intermolecular interactions by LigPlot+.
Figure S12. Best structure from the best and most populated Haddock cluster for the EphA2-Sam/D674N SASH1-Sam1 complex and summary of intermolecular interactions by LigPlot+.

Sequence 1: Homo Sapiens EphA2-Sam
Sequence 2: Mus musculus EphA2-Sam

```
95.9% identity in 73 residues overlap; Score: 363.0; Gap frequency: 0.0%

Sequence1      1 VPFRTVSEWLESIKMQQYTEHFMAAGYTAIEKVVQMTNDDIKRIGVRLPGHQKRIAYSLL
Sequence2      1 VPFRTVSEWLESIKMQQYTEHFMVAGYTAIEKVVQMSNEDIKRIGVRLPGHQKRIAYSLL
                 *********************** ************ * *********************

Sequence1     61 GLKDQVNTVGIPI
Sequence2     61 GLKDQVNTVGIPI
                 *************
```

Sequence 1: Homo sapiens EphA2-Sam
Sequence 2: Mus musculus EphA8-Sam

```
38.2% identity in 68 residues overlap; Score: 151.0; Gap frequency: 0.0%

Sequence1      5 TVSEWLESIKMQQYTEHFMAAGYTAIEKVVQMTNDDIKRIGVRLPGHQKRIAYSLLGLKD
Sequence2      2 TVGDWLDSIRMGRYRDHFAAGGYSSLGMVLRMNAQDVRALGITLMGHQKKILGSIQTMRA
                 **  ** ** *  *  ** * **     *  *   *    *  * **** *  *

Sequence1     65 QVNTVGIP
Sequence2     62 QLSSTQGP
                 *      *
```

Sequence 1: Homo sapiens EphA2-Sam
Sequence 2: Mus musculus EphA4-Sam

```
43.5% identity in 62 residues overlap; Score: 152.0; Gap frequency: 0.0%

Sequence1      5 TVSEWLESIKMQQYTEHFMAAGYTAIEKVVQMTNDDIKRIGVRLPGHQKRIAYSLLGLKD
Sequence2      2 SVGDWLQAIKMDRYKDNFTAAGYTTLEAVVHMSQDDLARIGITAITHQNKILSSVQAMRT
                  *  **  ***  *   * *****  * ** *  **  ***     **  *  *

Sequence1     65 QV
Sequence2     62 QM
                 *
```

Sequence 1: Mus musculus EphA2-Sam
Sequence 2: Mus musculus EphA8-Sam

```
36.8% identity in 68 residues overlap; Score: 150.0; Gap frequency: 0.0%

Sequence1      5 TVSEWLESIKMQQYTEHFMVAGYTAIEKVVQMSNEDIKRIGVRLPGHQKRIAYSLLGLKD
Sequence2      2 TVGDWLDSIRMGRYRDHFAAGGYSSLGMVLRMNAQDVRALGITLMGHQKKILGSIQTMRA
                 **  ** ** *  *  **   **     *  *   *    *  * **** *  *

Sequence1     65 QVNTVGIP
Sequence2     62 QLSSTQGP
                 *      *
```

Sequence 1: Mus musculus EphA2-Sam
Sequence 2: Mus musculus EphA4-Sam

```
41.9% identity in 62 residues overlap; Score: 147.0; Gap frequency: 0.0%

Sequence1      5 TVSEWLESIKMQQYTEHFMVAGYTAIEKVVQMSNEDIKRIGVRLPGHQKRIAYSLLGLKD
Sequence2      2 SVGDWLQAIKMDRYKDNFTAAGYTTLEAVVHMSQDDLARIGITAITHQNKILSSVQAMRT
                  *  **  ***  *   *  ****  * ** **  *  ***     **  *  *

Sequence1     65 QV
Sequence2     62 QM
                 *
```

Figure S1. Sequence identity assessment: EphA2 (Homo sapiens) vs EphA2 (Mus musculus)/EphA8 (Mus musculus)/ EphA4 (Mus musculus) and EphA2 (Mus musculus) vs EphA8 (Mus musculus)/EphA4 (Mus musculus). The alignments and percentages of identity represent outputs from the SIM [1] – Alignment Tool for Protein Sequences in EXPASY [2] (https://web.expasy.org/sim/, access date 13 December 2024).

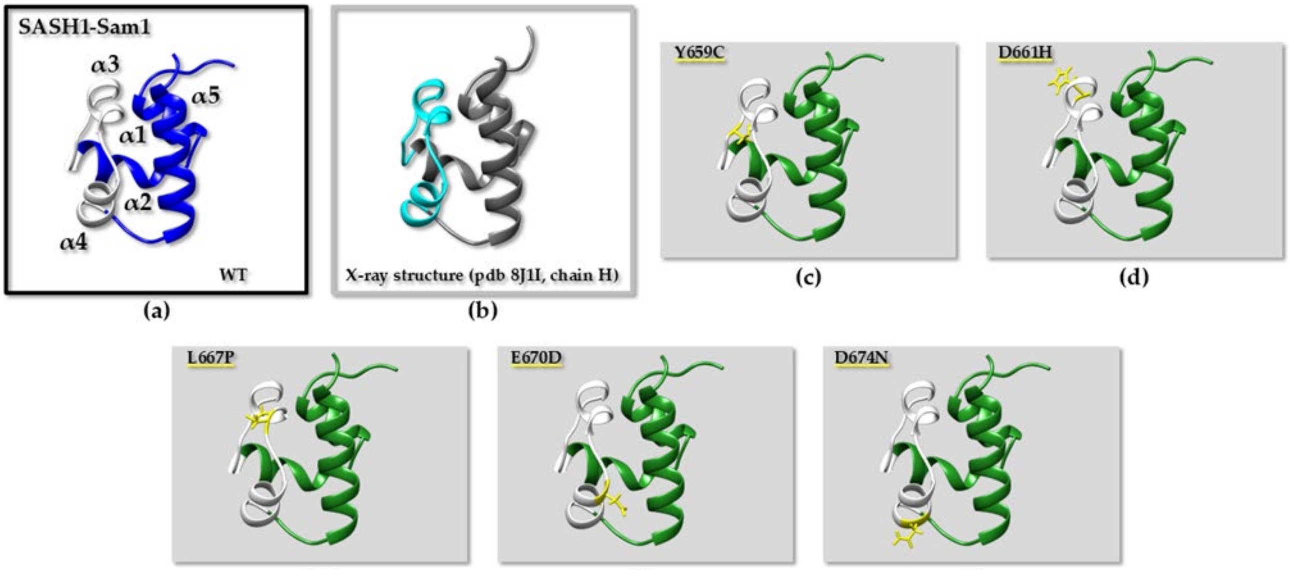

(a) (b) (c) (d) (e) (f) (g)

Figure S2. (a) AlphaFold2 (AF2) [3, 4] model of SASH1-Sam1 (blue). The human SASH1 WT sequence retrieved from UniProtKB [5] (code O94885, residue range P632-Y697, encompassing the Sam1 domain including a.a. from P633 to Y697) was employed to generate the AF2 prediction. The ML Interface is highlighted in white (residues from F656 to L676). (**b**) SASH1-Sam1 X-ray structure (grey) (pdb entry code 8J1I [6], chain H, Mus Musculus sequence residues P625-Y690) with the ML Interface colored cyan (residue range F649-L669). (**c-g**) AF2 models [3, 4] of diverse human SASH1-Sam1 cancer-associated variants (green) carrying point mutations positioned inside the ML Interface (white): Y659C (**c**), D661H (**d**), L667P (**e**), E670D (**f**) and D674N (**g**). The side chains of mutated residues are shown in yellow (**c-g**).

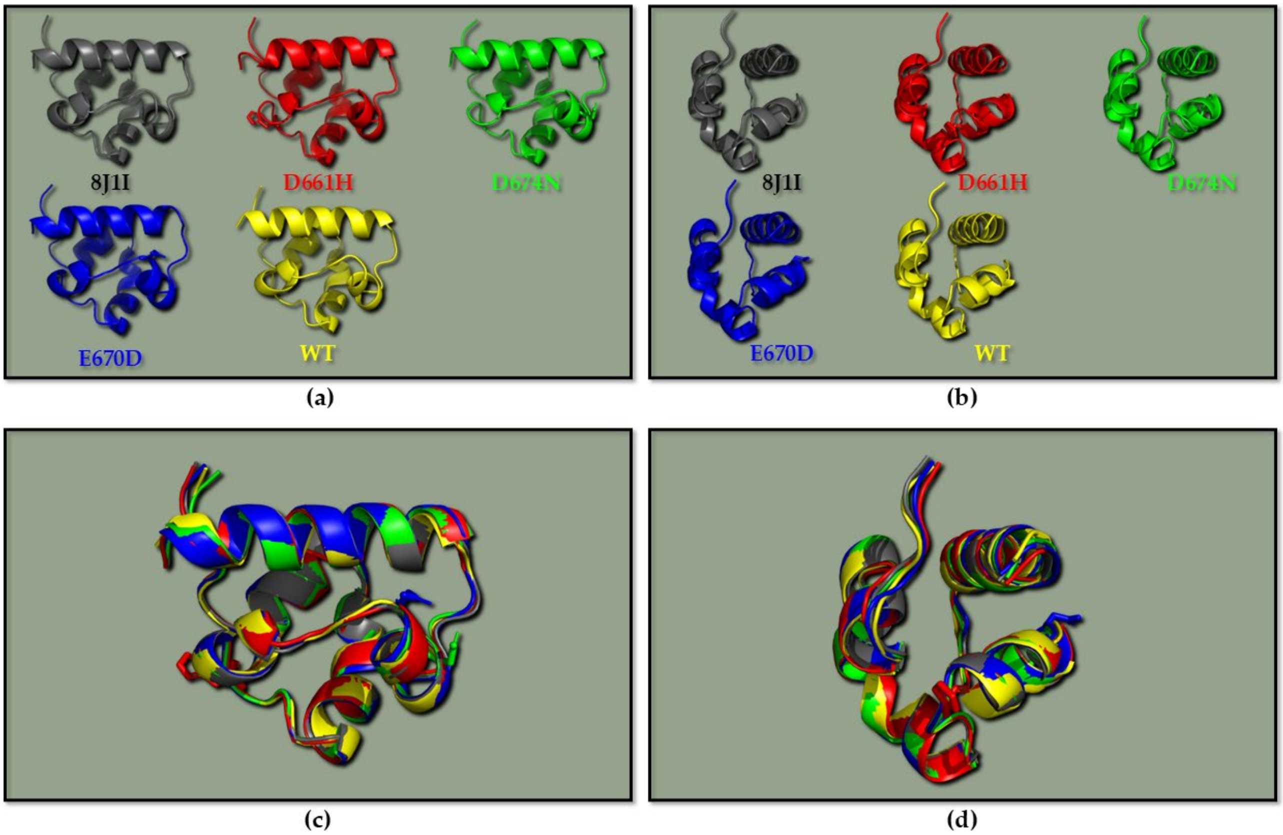

Figure S3. (**a, b**) The representative structures, extracted from the MD simulations, of diverse SASH1-Sam1 variants (i.e., human SASH1-Sam1 (WT) (yellow); Mouse SASH1-Sam1 (8J1I) (black) and cancer-related mutants: D661H (red), D674N (green), E670D (blue), are shown in cartoon representations as separate models. Two different orientations are presented in (**a**) and (**b**). (**c, d**) Corresponding overlays on the Cα atoms of all models are reported in the lower panels.

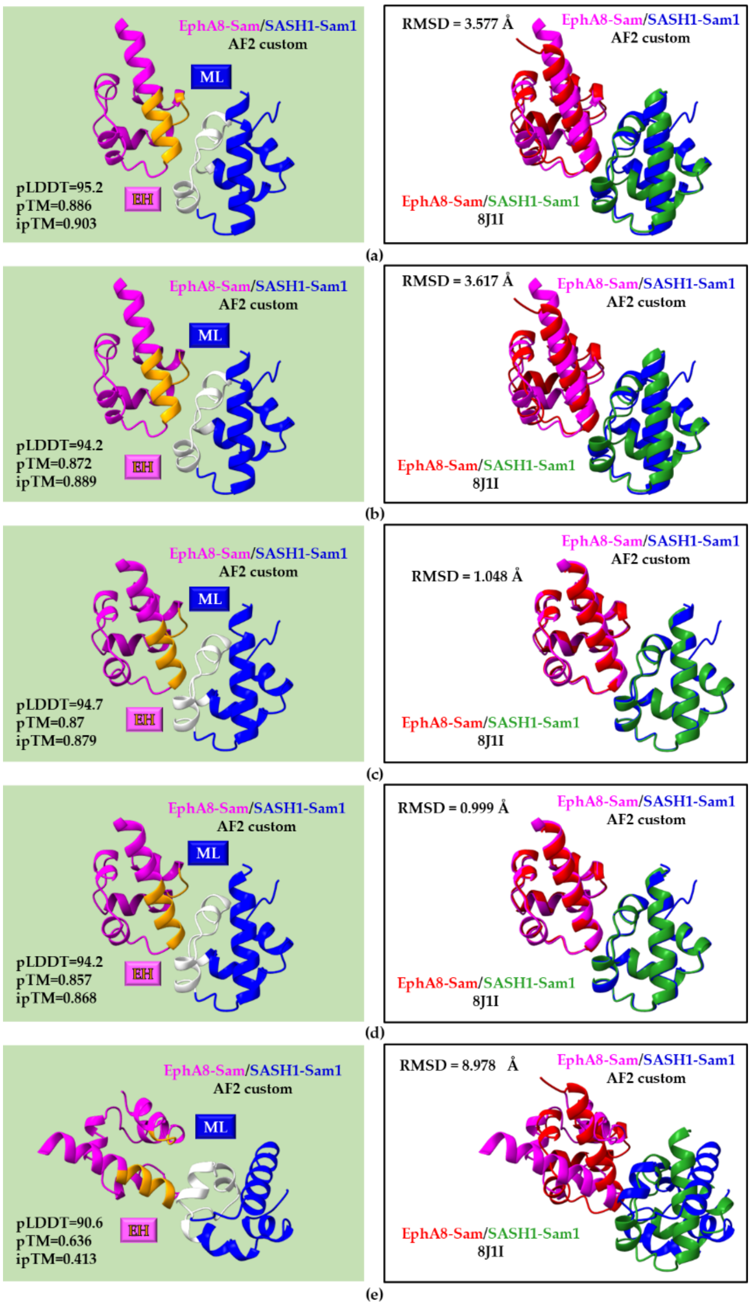

Figure S4. (**a-e, Left**) AF2 [3, 4] predicted models of Mus musculus EphA8-Sam (UniProtKB [5] entry O09127, residues T933–S996) in complex with Mus musculus SASH1-Sam1 (UniProtKB [5] entry P59808, residues P625-Y690). The 1st, 2nd, 3rd, 4th and 5th best predictions are reported in panels (**a**), (**b**), (**c**), (**d**), (**e**), respectively, with associated pLDDT, pTM, and ipTM scores [7, 8]. The predictions are based on the "custom" option of the "template_mode" parameter by using the pdb structure 8J1I [6] as custom template. EphA8-Sam and its EH region (i.e., residues I941-M943 and M977-G985) are colored in magenta and orange, respectively. SASH1-Sam1 and its ML region (i.e., residues F649-L669) are colored in blue and white, respectively. (**a-e, Right**) Superimposition on the backbone atoms of the X-ray structure of the EphA8-Sam/SASH1-Sam1 complex (pdb entry code 8J1I [6]) and the AF2 predicted models of the Mus musculus EphA8-Sam/SASH1-Sam1 complex. Overlays are shown for each of the best five predicted AF2 models. The backbone (bb) atoms (i.e., N, Cα and C') of residues T933-S996 (EphA8-Sam, pdb entry 8J1I, chain 8) and P625-Y690 (SASH1-Sam1, pdb entry 8J1I, chain H) in the crystal structure of the EphA8-Sam/SASH1-Sam1 complex were superimposed on the bb atoms of the corresponding residues in the AF2 models of the complex between EphA8-Sam and SASH1-Sam1. The adopted color code for the X-ray complex is: red for EphA8-Sam and dark green for SASH1-Sam1. The color code for the AF2 models is: magenta for EphA8-Sam and blue for SASH1-Sam1.

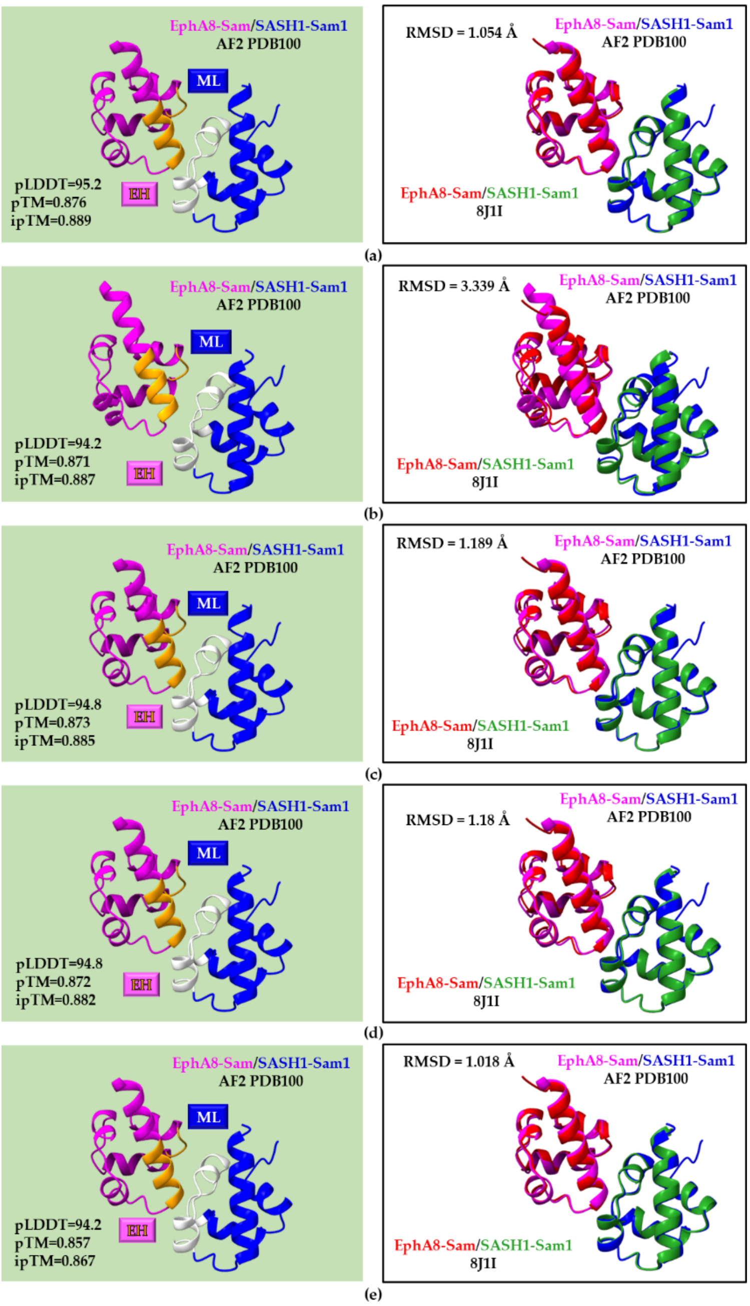

Figure S5. (**a-e, Left**) AF2 [3, 4] predicted models of Mus musculus EphA8-Sam (UniProtKB [5] entry O09127 residues T933–S996) in complex with Mus musculus SASH1-Sam1 (UniProtKB [5] entry P59808, residues P625-Y690). The 1st, 2nd, 3rd, 4th and 5th best predictions are reported in panels (**a**), (**b**), (**c**), (**d**), (**e**), respectively, with associated pLDDT, pTM, and ipTM scores [7, 8]. The predictions are based on the "PDB100" option of the "template_mode" parameter. The EphA8-Sam and its EH region (i.e., residues I941-M943 and M977-G985) are colored in magenta and orange, respectively. SASH1-Sam1 and its ML region (i.e., residues F649-L669) are colored in blue and white, respectively. (**a-e, Right**) Superimposition on the backbone atoms of the X-ray structure of the EphA8-Sam/SASH1-Sam1 complex (pdb entry code 8J1I [6]) and the AF2 predicted models of the Mus musculus EphA8-Sam/SASH1-Sam1 complex. Overlays are shown for each of the best five predicted AF2 models. The backbone (bb) atoms (i.e., N, Cα and C') of residues T933-S996 (EphA8, pdb entry 8J1I, chain 8) and P625-Y690 (SASH1, pdb entry 8J1I, chain H) in the crystal structure of the EphA8-Sam/SASH1-Sam1 complex were superimposed on the bb atoms of the corresponding residues in the AF2 models of the complex between EphA8-Sam and SASH1-Sam1. The adopted color code for the X-ray complex is: red for EphA8-Sam and dark green for SASH1-Sam1. The color code for the AF2 models is: magenta for EphA8-Sam and blue for SASH1-Sam1.

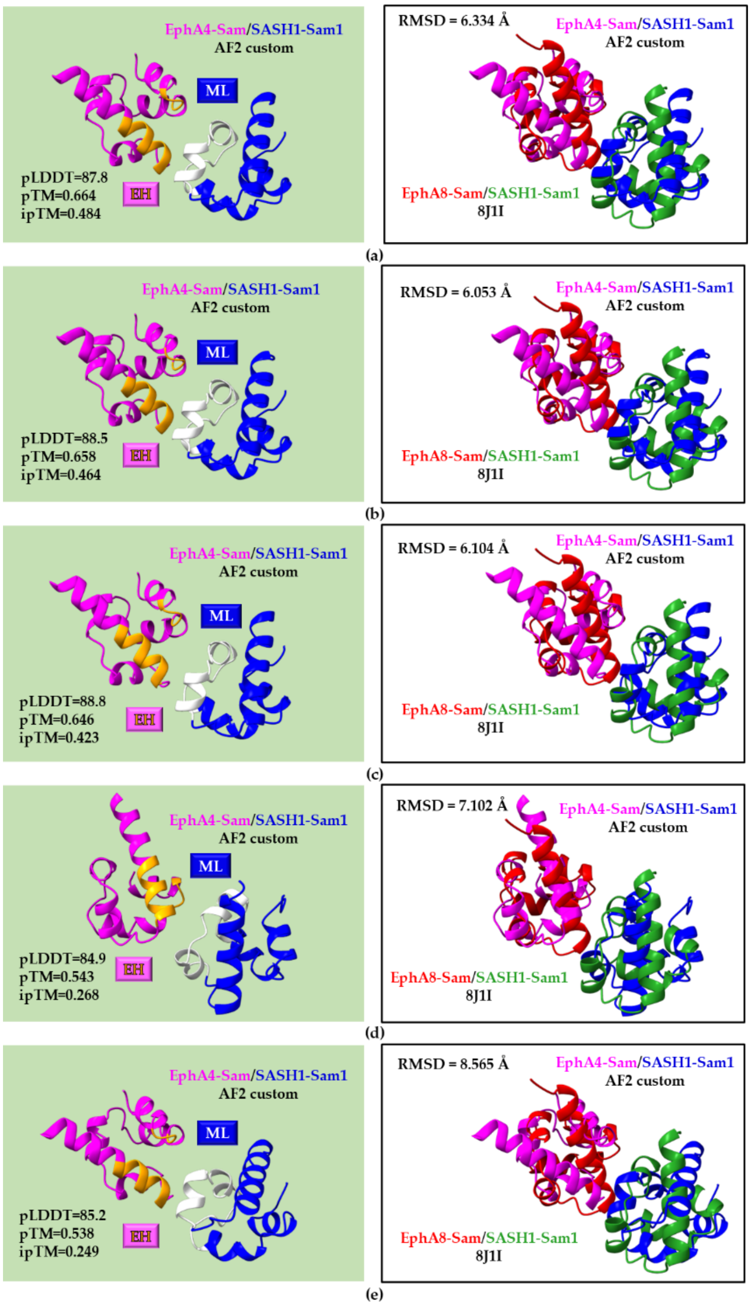

Figure S6. (**a-e**, **Left**) AF2 [3, 4] models of the Mus musculus EphA4-Sam domain (UniProtKB [5] entry Q03137, residues S915-Q978) in complex with the Mus musculus SASH1-Sam1 domain (UniProtKB [5] entry P59808, residues P625-Y690). The 1st, 2nd, 3rd , 4th and 5th best predictions are reported in panels (**a**), (**b**), (**c**), (**d**), (**e**), respectively, with associated pLDDT, pTM, and ipTM scores [7, 8]. The predictions are based on the "custom" option of the "template_mode" parameter by using the pdb structure 8J1I [6] as custom template. EphA4-Sam and its EH region (i.e., residues I923-M925 and I959-S967) are colored in magenta and orange, respectively. SASH1-Sam1 and its ML region (i.e., residues F649-L669) are colored in blue and white, respectively. (**a-e**, **Right**) Superimposition on the backbone atoms of the X-ray structure of the EphA8-Sam/SASH1-Sam1 complex (pdb entry code 8J1I [6]) and the AF2 predicted models of the Mus musculus EphA4-Sam/SASH1-Sam1 complex. Overlays are shown for each of the best five predicted AF2 models. The backbone (bb) atoms (i.e., N, Cα and C') of residues T933-S996 (EphA8-Sam, pdb entry 8J1I, chain 8) and P625-Y690 (SASH1-Sam1, pdb entry 8J1I, chain H) in the crystal structure of the EphA8-Sam/SASH1-Sam1 complex were superimposed on the bb atoms of the corresponding residues in the AF2 models of the complex between EphA4-Sam and SASH1-Sam1. The adopted color code for the X-ray EphA8-Sam/SASH1-Sam1 complex is: red for EphA8-Sam and dark green for SASH1-Sam1. The color code for the AF2 models of the EphA4-Sam/SASH1-Sam1 complex is: magenta for EphA4-Sam and blue for SASH1-Sam1.

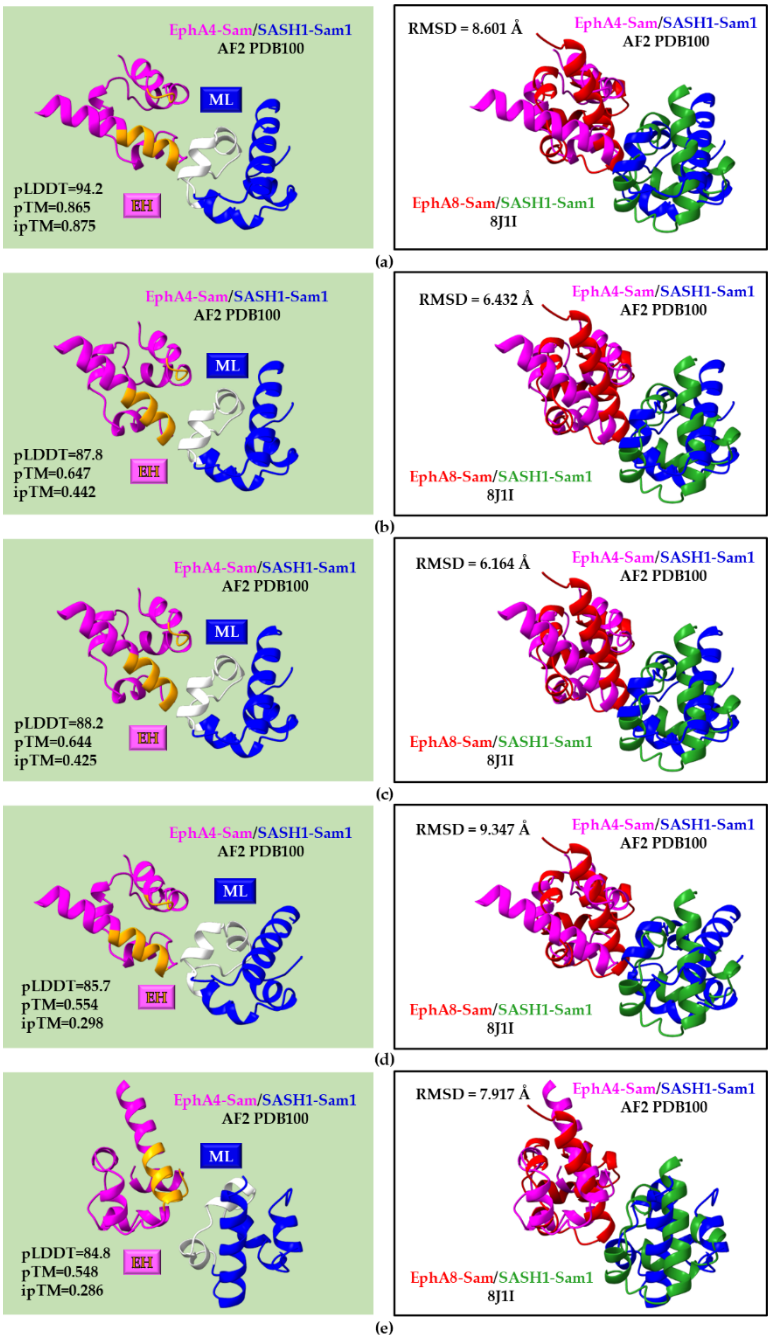

Figure S7. (**a-e**, **Left**) AF2 [3, 4] models of Mus musculus EphA4-Sam (UniProtKB [5] entry Q03137, residues S915-Q978) in complex with Mus musculus SASH1-Sam1 (UniProtKB [5] entry P59808, residues P625-Y690). The 1st, 2nd, 3rd , 4th and 5th best predictions are reported in panels (**a**), (**b**), (**c**), (**d**), (**e**), respectively, with associated pLDDT, pTM, and ipTM scores [7, 8]. The predictions are based on the "PDB100" option of the "template_mode" parameter. EphA4-Sam and its EH region (i.e., residues I923-M925 and I959-S967) are colored in magenta and orange, respectively. SASH1-Sam1 and its ML region (i.e., residues F649-L669) are colored in blue and white, respectively. (**a-e**, **Right**) Superimposition on the backbone atoms of the X-ray structure of the EphA8-Sam/SASH1-Sam1 complex (pdb entry code 8J1I [6]) and the AF2 predicted models of the Mus musculus EphA4-Sam/SASH1-Sam1 complex. Overlays are shown for each of the best five predicted AF2 models. The backbone (bb) atoms (i.e., N, Cα and C') of residues T933-S996 (EphA8-Sam, pdb entry 8J1I, chain 8) and P625-Y690 (SASH1-Sam1, pdb entry 8J1I, chain H) in the crystal structure of the EphA8-Sam/SASH1-Sam1 complex were superimposed on the bb atoms of the corresponding residues in the AF2 models of the complex between EphA4-Sam and SASH1-Sam1. The adopted color code for the EphA8-Sam/SASH1-Sam1 X-ray complex is: red for EphA8-Sam and dark green for SASH1-Sam1. The color code for the AF2 models of the EphA4-Sam/SASH1-Sam1 complex is: magenta for EphA4-Sam and blue for the SASH1-Sam1.

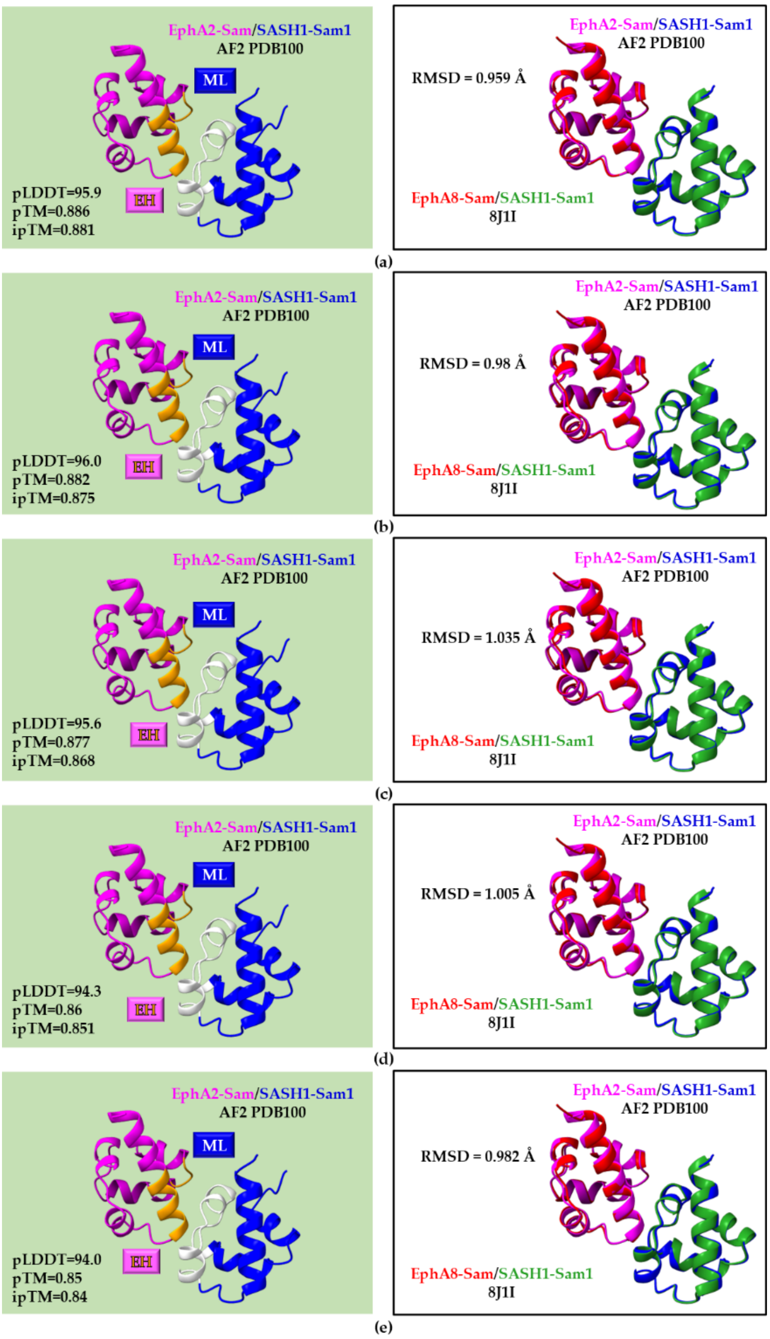

Figure S8. (**a-e, Left**) AF2 [3, 4] predicted models of human EphA2-Sam (UniProtKB [5] entry P29317, residues T908-T971) in complex with human SASH1-Sam1 (residues P632-Y697 from UniProtKB [5] entry O94885). The 1st, 2nd, 3rd, 4th and 5th best predictions are reported in panels (**a**), (**b**), (**c**), (**d**), (**e**), respectively, with associated pLDDT, pTM, and ipTM scores [7, 8]. The predictions were based on the "PDB100" option of the "template_mode" parameter. EphA2-Sam and its EH region (i.e., residues I916-M918 and P952-Y960) are colored in magenta and orange, respectively. SASH1-Sam1 and its ML region (i.e., residues F656-L676) are colored in blue and white, respectively. (**a-e, Right**) Superimposition on the backbone atoms of the X-ray structure of the EphA8-Sam/SASH1-Sam1 complex (pdb entry code 8J1I [6]) and the AF2 predicted models of the human EphA2-Sam/SASH1-Sam1 complex. Overlays are shown for each of the five best predicted AF2 models. The backbone (bb) atoms (i.e., N, Cα and C') of residues T933-S996 (EphA8-Sam, pdb entry 8J1I, chain 8) and P625-Y690 (SASH1-Sam1, pdb entry 8J1I, chain H) in the crystal structure of the EphA8-Sam/SASH1-Sam1 complex were superimposed on the bb atoms of the corresponding residues in the AF2 models of the complex between EphA2-Sam and SASH1-Sam1. The adopted color code for the EphA8-Sam/SASH1-Sam1 X-ray complex is: red for EphA8-Sam and dark green for SASH1-Sam1. The color code for the AF2 models of the EphA2-Sam/SASH1-Sam1 complex is: magenta for EphA2-Sam and blue for the SASH1-Sam1.

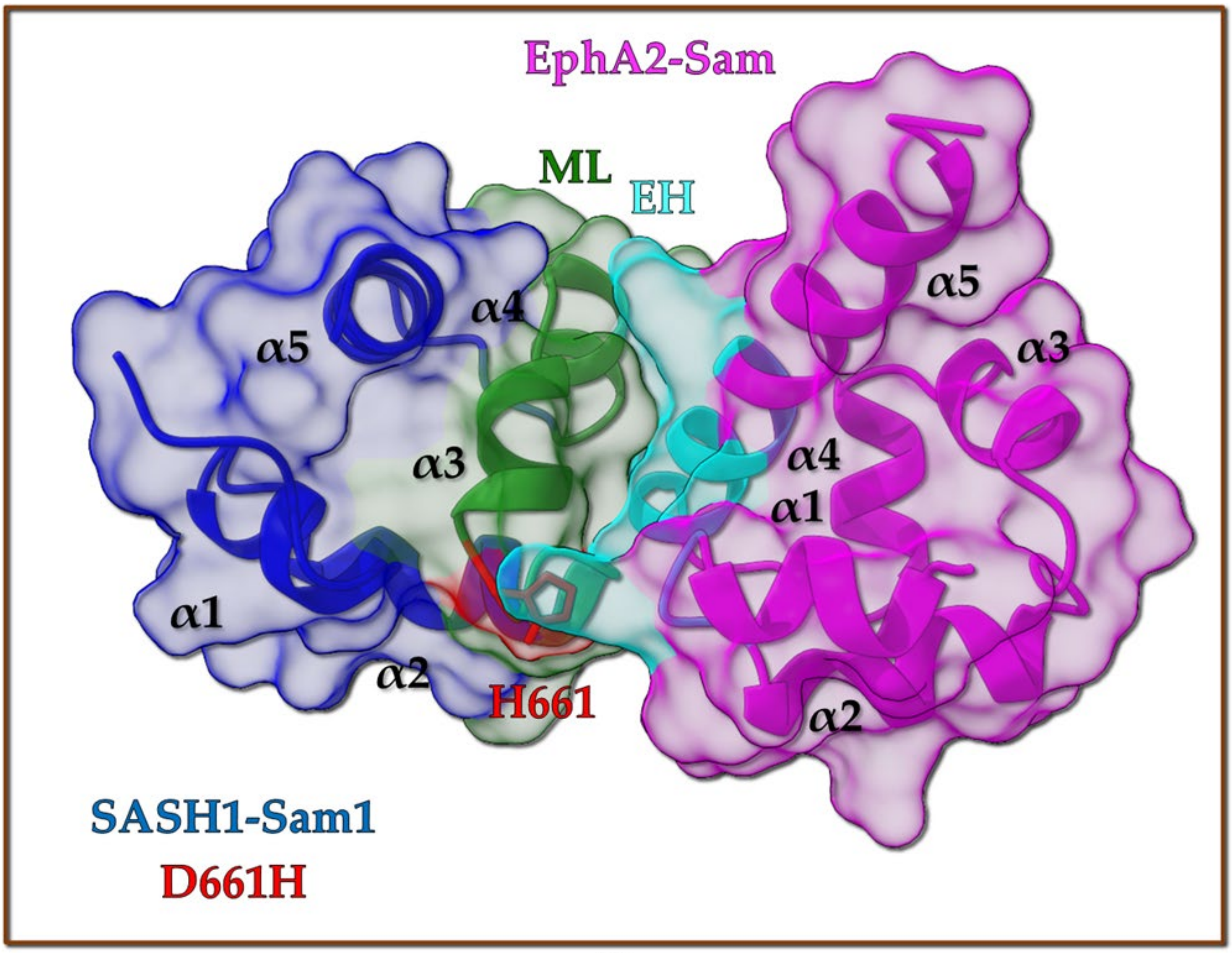

(a)

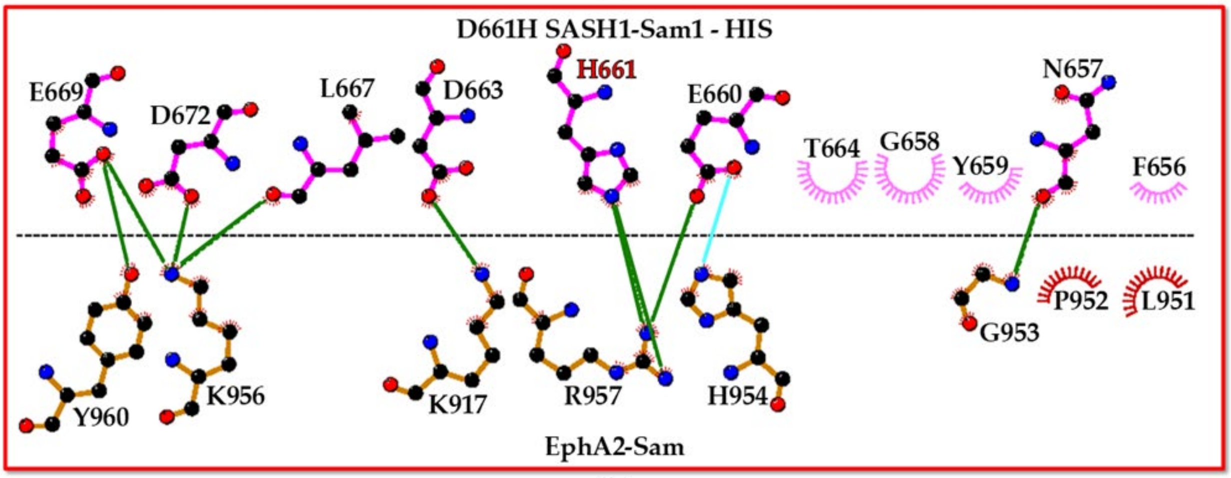

(b)

Figure S9. Best pose of the best and most populated Haddock [9] cluster of the EphA2-Sam/D661H SASH1-Sam1 complex. (**a**) The two Sam domains are shown in ribbon representation with a transparent surface. The blue and green colors indicate D661H SASH1-Sam1 and its ML region (residues F656-L676, sequence numbering according to UniprotKB [5] entry O94885 for human SASH1), respectively. The side chain of H661 ("HISD" protonation state) is shown in red. The magenta and cyan colors highlight EphA2-Sam and its EH region (I916-M918 and P952-Y960, sequence numbering according to UniprotKB [5] entry P29317 for human EphA2), respectively. (**b**) LigPlot+ [10, 11] analysis of the Sam-Sam interaction interface: black, blue, and red spheres refer to carbon, nitrogen, and oxygen atoms, respectively; green and cyan solid lines refer to H-bonds and salt bridges, respectively; non-bonded interactions are represented by the crescents with bristles. The Figure shown in (**a**) was produced with ChimeraX (version 1.5) [12].

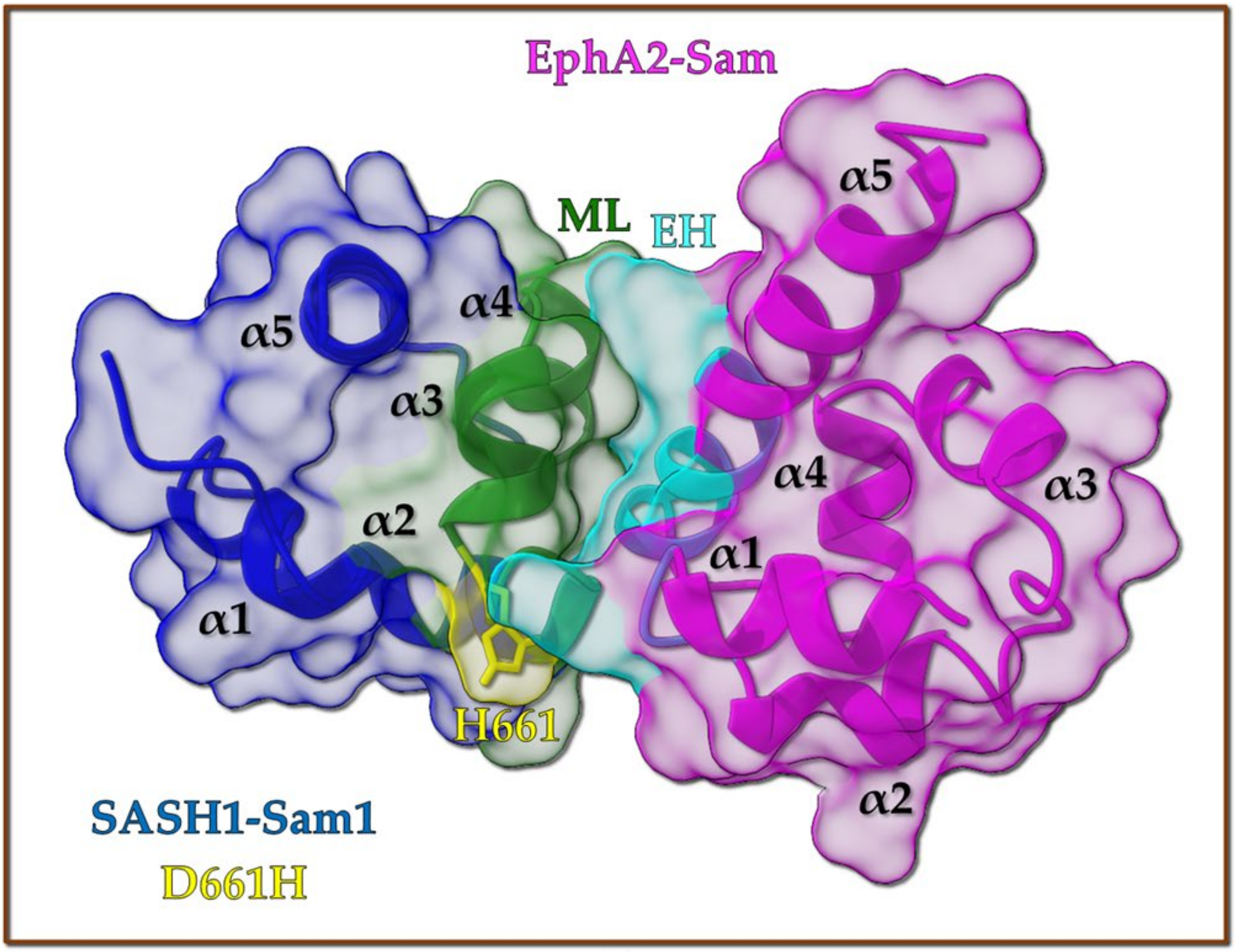

(a)

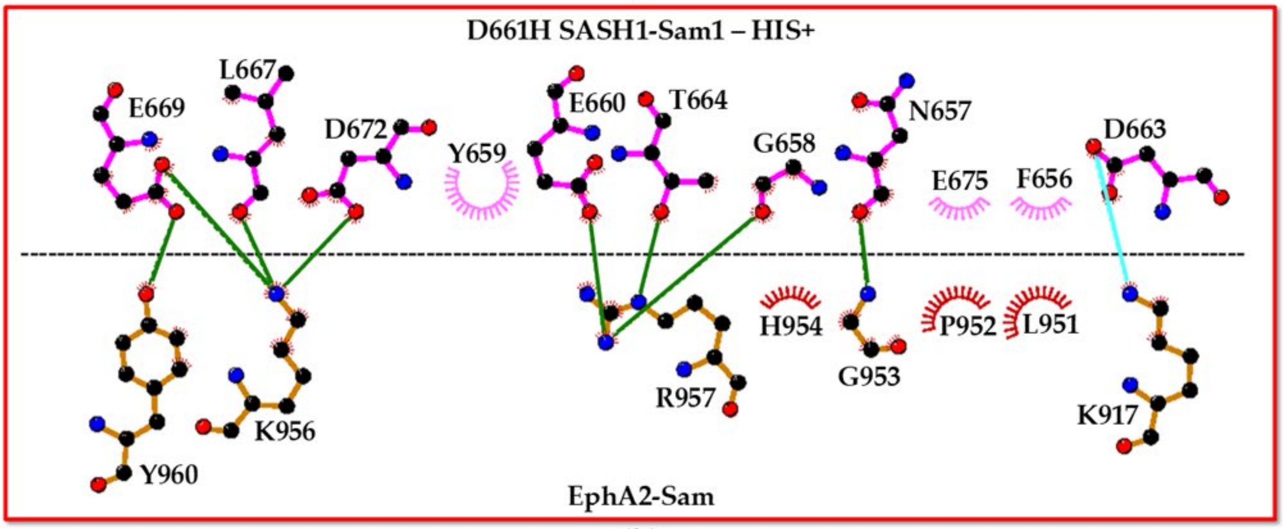

(b)

Figure S10. Best pose of the most populated Haddock [6] cluster of the EphA2-Sam/D661H SASH1-Sam1 complex. (**a**) The two Sam domains are shown in ribbon representation with a transparent surface. The blue and green colors indicate D661H SASH1-Sam1 and its ML region (residues F656-L676, sequence numbering according to UniprotKB [5] entry O94885 for human SASH1), respectively. The side chain of H661 ("HIS+" protonation state) is shown in dark goldenrod. The magenta and cyan colors highlight EphA2-Sam and its EH region (residues I916-M918 and P952-Y960, sequence numbering according to UniprotKB [5] entry P29317 for human EphA2), respectively. (**b**) LigPlot+ [10,11] analysis of the interaction interface: black, blue, and red spheres refer to carbon, nitrogen, and oxygen atoms, respectively; green and cyan solid lines refer to H-bonds and salt bridges, respectively; non-bonded interactions are represented by the crescents with bristles. The Figure shown in (**a**) was produced with ChimeraX (version 1.5) [12].

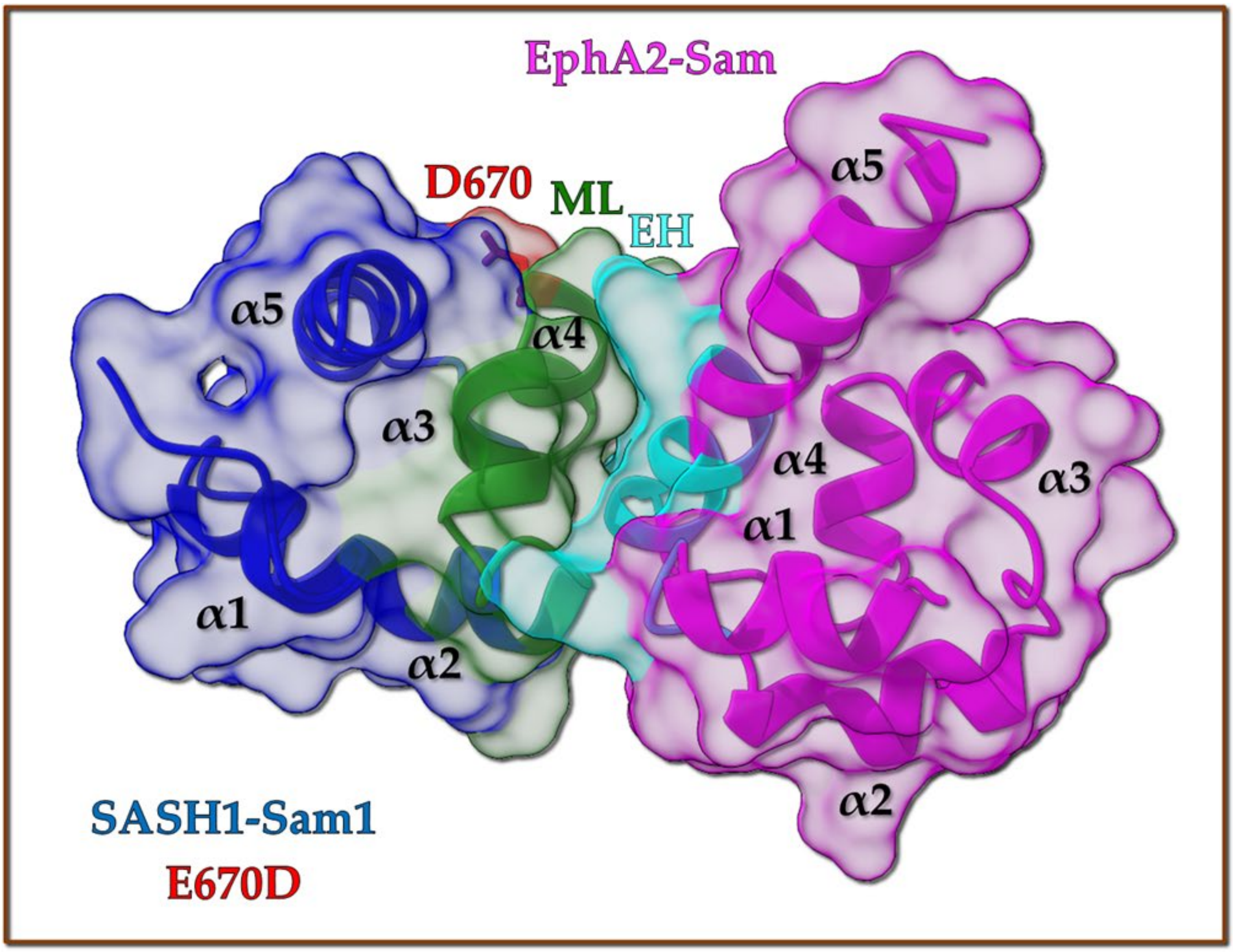

(a)

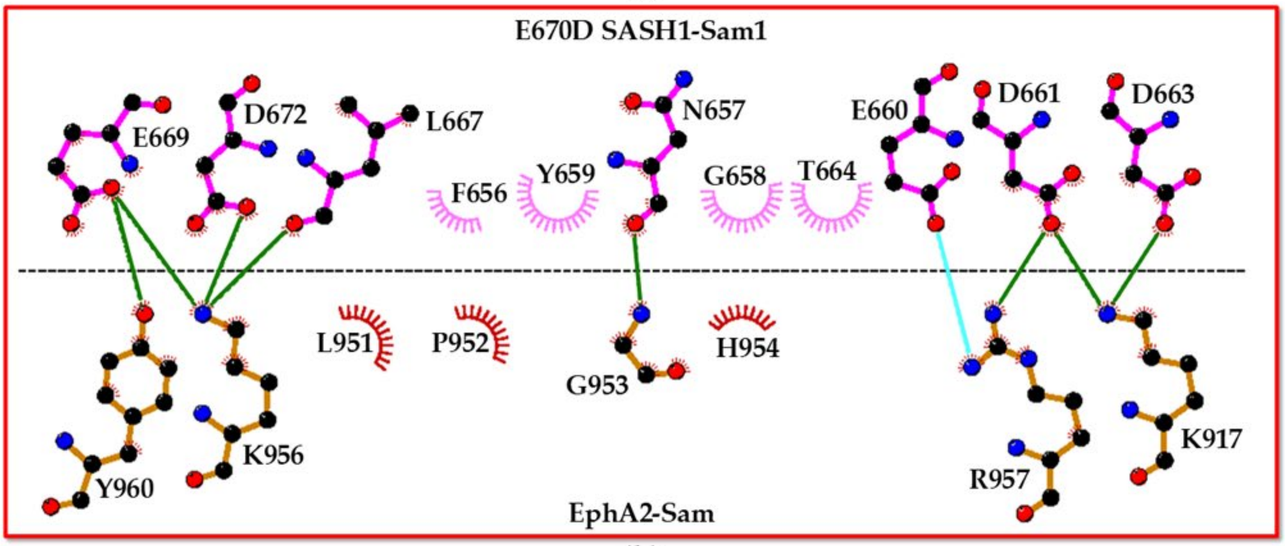

(b)

Figure S11. Best pose of the best and most populated Haddock [9] cluster of the EphA2-Sam/E670D SASH1-Sam1 complex. (**a**) The two Sam domains are shown in ribbon representation with a transparent surface. The blue and green colors indicate E670D SASH1-Sam1 and its ML region (residues F656-L676, sequence numbering according to UniprotKB [5] entry O94885 for human SASH1), respectively. The side chain of D670 is shown in red. The magenta and cyan colors highlight EphA2-Sam and its EH region (residues I916-M918 and P952-Y960, sequence numbering according to UniprotKB [5] entry P29317 for human EphA2), respectively. (**b**) LigPlot+ [10, 11] analysis of the interaction interface: black, blue, and red spheres refer to carbon, nitrogen, and oxygen atoms, respectively; green and cyan solid lines refer to H-bonds and salt bridges, respectively; non-bonded interactions are represented by the crescents with bristles. The Figure shown in (**a**) was produced with ChimeraX (version 1.5) [12].

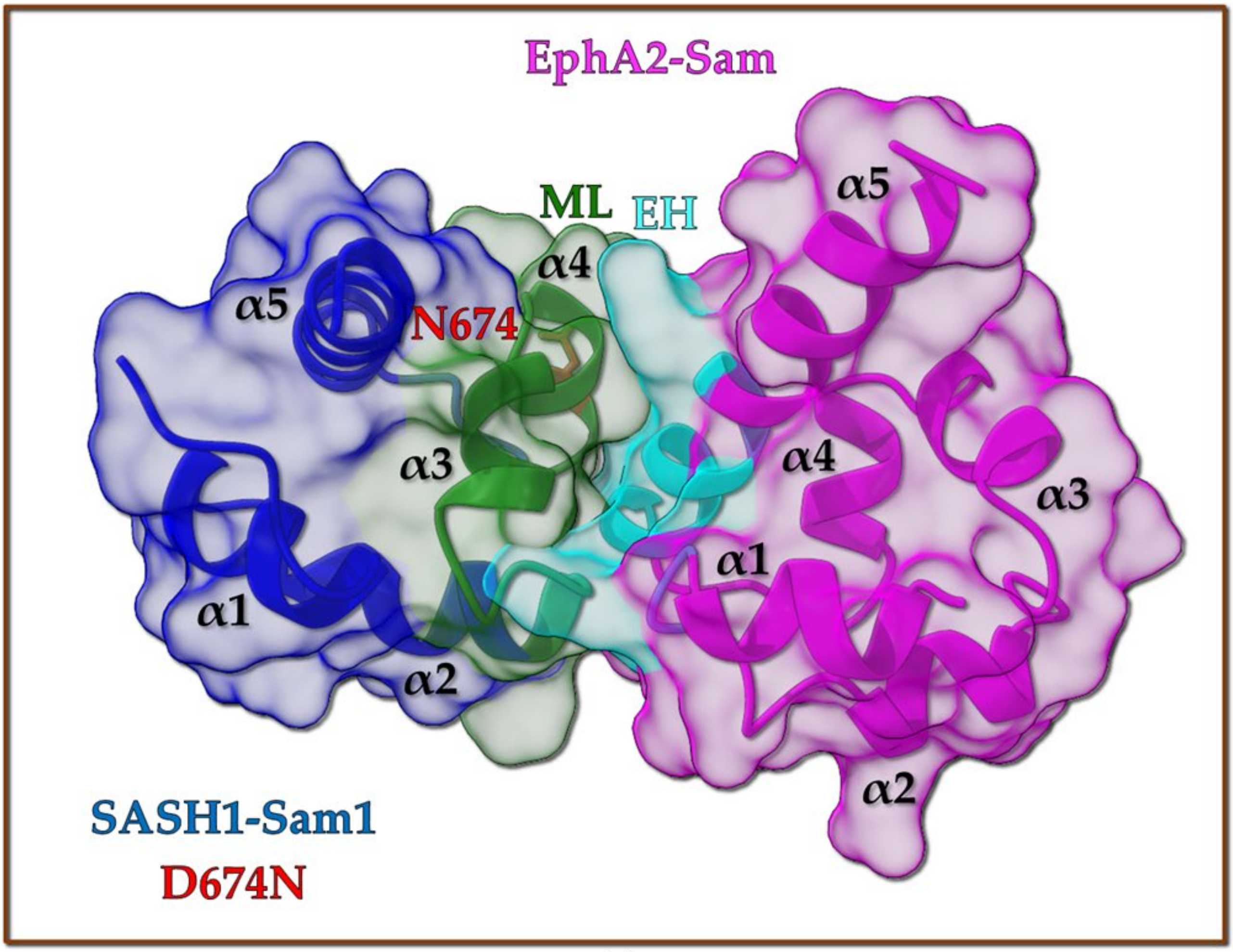

(a)

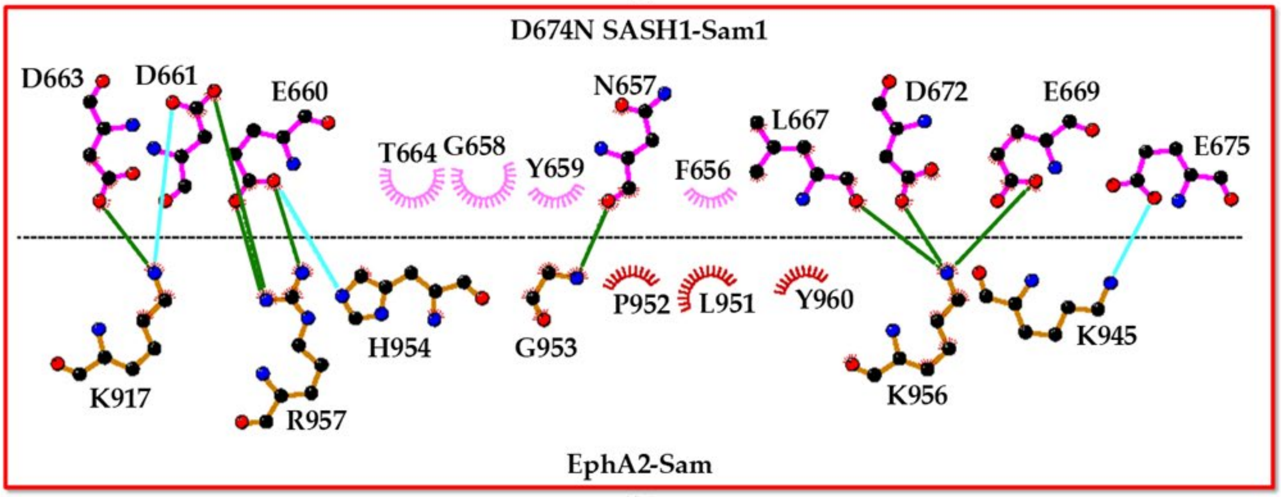

(b)

Figure S12. Best pose of the best and most populated Haddock [9] cluster of the EphA2-Sam/D674N SASH1-Sam1 complex. (**a**) The two Sam domains are shown in ribbon representation with a transparent surface. The blue and green colors indicate D674N SASH1-Sam1 and its ML region (residues F656-L676, sequence numbering according to UniprotKB [5] entry O94885 for human SASH1), respectively. The side chain of N674 is shown in red. The magenta and cyan colors highlight EphA2-Sam and its EH region (residues I916-M918 and P952-Y960, sequence numbering according to UniprotKB [5] entry P29317 for human EphA2), respectively. (**b**) LigPlot+ [10, 11] analysis of the interaction interface: black, blue, and red spheres refer to carbon, nitrogen, and oxygen atoms, respectively; green and cyan solid lines refer to H-bonds and salt bridges, respectively; non-bonded interactions are represented by the crescents with bristles. The Figure shown in (**a**) was produced with ChimeraX (Version 1.5) [12].

1. (=Reference 32 in the Main Text) Huang, X.; Miller, W. A Time-Efficient, Linear-Space Local Similarity Algorithm. *Adv. Appl. Math.* **1991,** *12*(3), 337-357.
2. (=Reference 33 in the Main Text) Wilkins, M.R.; Gasteiger, E.; Bairoch, A.; Sanchez, J.C.; Williams, K.L.; Appel, R.D.; Hochstrasser, D.F. Protein identification and analysis tools in the ExPASy server. *Methods Mol. Biol.* **1999,** *112*, 531-52.
3. (=Reference 53 in the Main Text) Jumper, J.; Evans, R.; Pritzel, A.; Green, T.; Figurnov, M.; Ronneberger, O.; Tunyasuvunakool, K.; Bates, R.; Zidek, A.; Potapenko, A.; Bridgland, A.; Meyer, C.; Kohl, S.A.A.; Ballard, A.J.; Cowie, A.; Romera-Paredes, B.; Nikolov, S.; Jain, R.; Adler, J.; Back, T.; Petersen, S.; Reiman, D.; Clancy, E.; Zielinski, M.; Steinegger, M.; Pacholska, M.; Berghammer, T.; Bodenstein, S.; Silver, D.; Vinyals, O.; Senior, A.W.; Kavukcuoglu, K.; Kohli, P.; Hassabis, D. Highly accurate protein structure prediction with AlphaFold. *Nature* **2021,** *596*(7873), 583-589.
4. (=Reference 54 in the Main Text) Varadi, M.; Anyango, S.; Deshpande, M.; Nair, S.; Natassia, C.; Yordanova, G.; Yuan, D.; Stroe, O.; Wood, G.; Laydon, A.; Zidek, A.; Green, T.; Tunyasuvunakool, K.; Petersen, S.; Jumper, J.; Clancy, E.; Green, R.; Vora, A.; Lutfi, M.; Figurnov, M.; Cowie, A.; Hobbs, N.; Kohli, P.; Kleywegt, G.; Birney, E.; Hassabis, D.; Velankar, S. AlphaFold Protein Structure Database: massively expanding the structural coverage of protein-sequence space with high-accuracy models. *Nucleic Acids Res.* **2022,** *50*(D1), D439-D444.
5. (=Reference 30 in the Main Text) UniProt Consortium. UniProt: the Universal Protein Knowledgebase in 2023. *Nucleic Acids Res.* **2023,** *51*(D1), D523-D531.
6. (=Reference 9 in the Main Text) Ding, Y.; Chen, Q.; Shan, H.; Liu, J.; Lv, C.; Wang, Y.; Yuan, L.; Chen, Y.; Wang, Z.; Yin, Y.; Xiao, K.; Li, J.; Liu, W. SASH1: A Novel Eph Receptor Partner and Insights into SAM-SAM Interactions. *J. Mol. Biol.* **2023,** *435*(19), 168243.
7. (=Reference 83 in the Main Text) Magana, P.; Kovalevskiy, O. pLDDT: Understanding local confidence. https://www.ebi.ac.uk/training/online/courses/alphafold/inputs-and-outputs/evaluating-alphafolds-predicted-structures-using-confidence-scores/plddt-understanding-local-confidence/ (accessed on 17/10/2024).
8. (=Reference 75 in the Main Text) Magana, P.; Kovalevskiy, O. Confidence scores in AlphaFold-Multimer. https://www.ebi.ac.uk/training/online/courses/alphafold/inputs-and-outputs/evaluating-alphafolds-predicted-structures-using-confidence-scores/confidence-scores-in-alphafold-multimer/ (accessed on 17/10/2024).
9. (=Reference 28 in the Main Text) de Vries, S.J.; van Dijk, M.; Bonvin, A.M. The HADDOCK web server for data-driven biomolecular docking. *Nat. Protoc.* **2010,** *5*(5), 883-97.
10. (=Reference 31 in the Main Text) Wallace, A.C.; Laskowski, R.A.; Thornton, J.M. LIGPLOT: a program to generate schematic diagrams of protein-ligand interactions. *Protein Eng.* **1995,** *8*(2), 127-34.
11. (=Reference 11 in the Main Text) Laskowski, R.A.; Swindells, M.B. LigPlot+: multiple ligand-protein interaction diagrams for drug discovery. *J. Chem. Inf. Model.* **2011,** *51*(10), 2778-86.
12. (=Reference 101 in the Main Text) Meng, E.C.; Goddard, T.D.; Pettersen, E.F.; Couch, G.S.; Pearson, Z.J.; Morris, J.H.; Ferrin, T.E. UCSF ChimeraX: Tools for structure building and analysis. *Protein Sci.* **2023,** *32*(11), e4792.
