# Supplementary material for: Sam-Sam Association Between EphA2 and SASH1: In Silico Studies of Cancer-Linked Mutations
Source: Molecules. 2025 Feb 5;30(3):718. doi: 10.3390/molecules30030718 (PMC11820823; doi:10.3390/molecules30030718)
Supplement: Supplementary file 1 [file molecules-30-00718-s001.zip › Supplementary Tables.pdf]

Supplementary Tables

Table S1. COSMIC missense mutations affecting SASH1-Sam1: conservation scores and solvent accessibilities for corresponding residues.

Table S2. Primary sequence analyses by EXPASY: evaluation of GRAVY, Theoretical pI, and Instability index values for human WT SASH1-Sam1 and cancer-associated mutants.

Table S3. RMSD values for the comparison of 3D coordinates of human WT and mouse SASH1-Sam1 domains.

Table S4. RMSD values for the comparison of 3D coordinates of human WT and mutated SASH1-Sam1 domains.

Table S5. HoTMuSiC output: predicted ΔTm values.

Table S6. RMSD values and hydrogen bonds along MD simulations for human and mouse wild-type SASH1-Sam1, indicated as "WT" and "8J1I", respectively and cancer-related variants.

Table S7. RMSD evaluation: the X-ray structure of the EphA8-Sam/SASH1-Sam1 complex (pdb entry 8J1I) vs each of the best five AF2 models of mouse EphA8-Sam/SASH1-Sam1, mouse EphA4-Sam/SASH1-Sam1 and human EphA2-Sam/SASH1-Sam1.

Tables S8 and S9. Validation of the in silico strategy relying on the Haddock Refinement Interface and the Prodigy webserver.

Table S10. Haddock refinement: number of clusters and relative populations obtained for the human EphA2-Sam/SASH1-Sam1 native and selected mutated complexes.

Table S11. Haddock scores for the human EphA2-Sam/SASH1-Sam1 native and mutated complexes.

Table S1. Evaluation of conservation scores and solvent accessibilities for SASH1-Sam1 residues related to COSMIC [1] missense mutations, obtained with ConSurf [2] and MolMol [3], respectively. Data refer to analyses of the best AF2 predicted model for human SASH1-Sam1 (UniprotKB [4] entry O94885, residue range from P632 to Y697). A probe radius equal to 1.4 Å and a precision equal to 5 were employed to measure the solvent accessible surface. Mutated amino acids belonging to the ML Interface (residue range from F656 to L676) are underlined.

SASH1-Sam1	ConSurf score	MolMol solvent accessibility (%)
P633*	9	45.0
R644	4	31.4
<u>Y659</u>	7	7.2
<u>D661</u>	6	31.2
<u>L667</u>	4	45.2
<u>E670</u>	5	28.8
<u>D674</u>	5	21.5
R679	1	46.4
P681	4	38.9
R684	9	10.7
V686	4	24.5
L687	6	0.0
L688	7	17.2

*P633 represents the first N-terminal residue in the pdb file and is thus associated with high solvent exposure.

Table S2. Analysis of primary sequences through the Protparam tool of ExPASy [5, 6] (https://web.expasy.org/protparam/, access date 12 September 2024). The following parameters were evaluated: GRAVY, Theoretical pI, and Instability index. The input amino acid sequences consist of human WT SASH1-Sam1 (residue range P632-Y697, UniprotKB [4] entry O94885 for human SASH1) and associated cancer mutants. Residues belonging to the ML Interface (from F656 to L676) have been underlined.

SASH1-Sam1	GRAVY	Theoretical pI	Instability index
WT	-0.570	4.25	49.52
P633L	-0.488	4.25	48.62
R644Q	-0.555	4.14	50.66
<u>Y659C</u>	-0.512	4.25	51.95
<u>D661H</u>	-0.565	4.42	45.46
<u>L667P</u>	-0.652	4.25	49.67
<u>E670D</u>	-0.570	4.22	42.56
<u>D674N</u>	-0.570	4.31	49.52
R679S	-0.514	4.14	49.52
P681L	-0.488	4.25	46.89
P681S	-0.558	4.25	52.72
R684S	-0.514	4.14	49.52
V686G	-0.639	4.25	49.52
L687I	-0.559	4.25	52.44
L688F	-0.585	4.25	49.52

Table S3. The 3D coordinates of human wild-type ("WT") and mouse ("8J1I") SASH1-Sam1 domains were compared by evaluating RMSD values with MolMol [3]. RMSD values were calculated by superimposing either all the backbone (bb) atoms (i.e., N, Cα and C') in residues from P632 to Y697 of human SASH1-Sam1 (UniProtKB [4] code O94885) with the corresponding residues from P625 to Y690 of mouse SASH1-Sam1 (pdb entry 8J1I [7], chain H) or just the bb atoms in the ML Interfaces (residues F656-L676 of human SASH1 and the corresponding residues F649-L669 of mouse SASH1).

	**SASH1-Sam1**	**RMSD (Å)**	
		**bb all**	**bb ML**
1	WT[@]	-----------	-----------
2	8J1I[#]	1.178	0.470

[@] The AF2 best model.

[#]The chain H of the PDB entry 8J1I [7].

Table S4. The 3D coordinates of human wild-type (WT) and mutated SASH1-Sam1 domains were compared through RMSD evaluation. RMSD values were calculated with MolMol [3] by overlaying on diverse atom sets (as reported in the various columns) the AF2 [8, 9] models of SASH1-Sam1 WT and the diverse mutated domains. "Heavy all" and "bb" stand for all the heavy atoms (C, N, O, S) and backbone atoms (N, Cα and C'), respectively, in all residues from P632 to Y697 (SASH1-Sam1) but not including those in mutated side chains.

	**SASH1-Sam1[@]**	**RMSD (Å)**		
		**bb all**	**Heavy all**	**bb ML**
1	WT	-----------	-----------	-----------
2	Y659C*	0.377	0.832	0.422
3	D661H	0.129	0.868	0.159
4	L667P	0.278	0.797	0.293
5	E670D	0.066	0.519	0.065
6	D674N	0.116	0.641	0.077

*Only mutations positioned inside the ML interface of SASH1-Sam1 were analyzed.

[@]AF2 best models.

Table S5. Results from HoTMuSiC [10, 11] analysis: ΔTm estimates for cancer-linked mutations inside the SASH1-Sam1 domain. Mutations affecting the ML Interface are underlined. Destabilizing mutations are associated with predicted values <-1 and are highlighted red.

SASH1-Sam1 WT[@]	HoTMuSiC ΔTm (°C)
P633L	-2.39
R644Q	-0.63
Y659C	-6.51
D661H	-1.29
L667P	-5.26
E670D	-2.65
D674N	-1.09
R679S	-1.88
P681L	-2.36
P681S	-3.86
R684S	-7.48
V686G	-5.03
L687I	-6.47
L688F	-5.33

[@] The best AF2 model of wild-type SASH1-Sam1 was employed as input for the analysis.

Table S6. RMSD values computed along the MD simulations considering the Cα atoms for the human and mouse wild-type SASH1-Sam1 domains (indicated as "WT" and "8J1I") and diverse cancer-associated variants.

System	RMSD (nm)		Number of Hydrogen bonds	
	Standard Deviations	Mean values	Standard Deviations	Mean values
**8J1I**	0.03	0.16	3.9	50.1
**D661H**	0.02	0.14	3.9	51.0
**D674N**	0.02	0.12	3.8	50.1
**E670D**	0.02	0.13	4.0	50.3
**WT**	0.04	0.15	3.9	50.1

Table S7. Comparison between the coordinates of the EphA8-Sam/SASH1-Sam1 complex (X-ray structure, pdb entry 8J1I [7] chains 8 and H) and each of the best five AF2 [8, 9] models obtained for the following complexes: mouse EphA8-Sam/SASH1-Sam1, mouse EphA4-Sam/SASH1-Sam1 and human EphA2-Sam/SASH1-Sam1. The RMSD values were calculated with MolMol [3] by superimposing the backbone (bb) atoms (i.e., N, Cα and C') of the EphA8-Sam/SASH1-Sam1 X-ray structure [residues T933-S996 of mouse EphA8 (chain 8) and P625-Y690 of mouse SASH1 (chain H)], to the bb atoms of the corresponding residues in the AF2 models of the complex between mouse EphA8-Sam (i.e., residues T933-S996, according to the UniProtKB [4] code O09127) and SASH1-Sam1 (residues P625-Y690, according to the UniProtKB [4] code P59808); the complex between mouse EphA4-Sam (residues S915-Q978, according to the UniProtKB [4] code Q03137) and SASH1-Sam1; the complex between human EphA2-Sam (residues T908-T971, according to the UniProtKB [4] code P29317) and SASH1-Sam1 (residues P632-Y697, according to the UniProtKB [4] code O94885).

**Structure**	**RMSD (Å)**
	**bb** **all**
8J1I	-----------
1st EphA8-Sam/SASH1-Sam1 "custom"	3.577
2nd EphA8-Sam/SASH1-Sam1 "custom"	3.617
3rd EphA8-Sam/SASH1-Sam1 "custom"	1.048
4th EphA8-Sam/SASH1-Sam1 "custom"	0.999
5th EphA8-Sam/SASH1-Sam1 "custom"	8.978
1st EphA8-Sam/SASH1-Sam1 "PDB100"	1.054
2nd EphA8-Sam/SASH1-Sam1 "PDB100"	3.339
3rd EphA8-Sam/SASH1-Sam1 "PDB100"	1.189
4th EphA8-Sam/SASH1-Sam1 "PDB100"	1.18
5th EphA8-Sam/SASH1-Sam1 "PDB100"	1.018
1st EphA4-Sam/SASH1-Sam1 "custom"	6.334
2nd EphA4-Sam/SASH1-Sam1 "custom"	6.053
3rd EphA4-Sam/SASH1-Sam1 "custom"	6.104
4th EphA4-Sam/SASH1-Sam1 "custom"	7.102
5th EphA4-Sam/SASH1-Sam1 "custom"	8.565
1st EphA4-Sam/SASH1-Sam1 "PDB100"	8.601
2nd EphA4-Sam/SASH1-Sam1 "PDB100"	6.432
3rd EphA4-Sam/SASH1-Sam1 "PDB100"	6.164
4th EphA4-Sam/SASH1-Sam1 "PDB100"	9.347
5th EphA4-Sam/SASH1-Sam1 "PDB100"	7.917
1st EphA2-Sam/SASH1-Sam1 "PDB100"	0.959
2nd EphA2-Sam/SASH1-Sam1 "PDB100"	0.98
3rd EphA2-Sam/SASH1-Sam1 "PDB100"	1.035
4th EphA2-Sam/SASH1-Sam1 "PDB100"	1.005
5th EphA2-Sam/SASH1-Sam1 "PDB100"	0.982

Table S8. Validation of the computational protocol. Outputs (including number of clusters and corresponding populations) of the Haddock Refinement Interface [12] for the mouse EphA8-Sam/SASH1-Sam1 wild-type (wt) and the EphA8-Sam/Y652A SASH1-Sam1 mutated complexes. The input structure for the Haddock refinements consisted of the AF2 model of wt mus musculus EphA8-Sam (residues L932–L1004 in UniProtKB [4] entry O09127) in complex with SASH1-Sam1 (residues P625-D691 in UniProtKB [4] entry P59808). The Haddock scores were employed to rank the diverse clusters: the 1st cluster represents the one with the best (i.e., lowest) Haddock score, calculated as average value over the best 4 structures of the cluster [12].

EphA8-Sam	SASH1-Sam1	Cluster	Population
wt	wt	1st<br>Haddock cluster 1	33/90
		2nd<br>Haddock cluster 6	5/90
		3rd<br>Haddock cluster 3	18/90
		4th<br>Haddock cluster 4	6/90
		5th<br>Haddock cluster 5	6/90
		6th<br>Haddock cluster 2	22/90
wt	Y652A	1st<br>Haddock cluster 1	35/90
		2nd<br>Haddock cluster 3	22/90
		3rd<br>Haddock cluster 4	9/90
		4th<br>Haddock cluster 2	24/90

Table S9. Validation of the computational protocol. Haddock scores [12] and dissociation constant values by PRODIGY [13] were evaluated for the mouse wt EphA8-Sam/SASH1-Sam1 and the EphA8-Sam/Y652A SASH1-Sam1 complexes as average values over the best 10 structures (IV and V columns) of the specified clusters or just for the best structure (VI and VII columns) of the cluster. Experimentally determined K_D (K_D exp) [7] values are reported in the last column.

EphA8-Sam	SASH1-Sam1	Cluster	Average Haddock Score	Average K_D (μM)	Haddock Score	K_D (μM)	K_D exp (μM)
wt	wt	1st (Best & Most populated)<br>Haddock cluster 1	-112 ± 5	10 ± 3	-124.3	7.8	0.88
wt	Y652A	1st (Best & Most populated)<br>Haddock cluster 1	-99 ± 2	14 ± 3	-104.3	9.0	~885

Table S10. Number of clusters and relative populations obtained through the Haddock refinement Interface [12] for the EphA2-Sam/SASH1-Sam1 native and selected mutated complexes. EphA2-Sam residues correspond to a.a. from T908 to V972 of human EphA2 (UniprotKB [4] entry P29317), for SASH1-Sam1 the residue range is P632-Y697 (UniprotKB [4] entry O94885 for human SASH1). For the D661H SASH1-Sam1 mutant the mutated histidine was set in both the uncharged (HISD) and positively charged (HIS+) states.

EphA2-Sam	SASH1-Sam1	Cluster	Population
wt	wt	1st Haddock cluster 1	61/87
		2nd Haddock cluster 2	16/87
		3rd Haddock cluster 3	10/87
wt	D661H (HISD)	1st Haddock cluster 1	59/90
		2nd Haddock cluster 2	19/90
		3rd Haddock cluster 3	8/90
		4th Haddock cluster 4	4/90
wt	D661H (HIS+)	1st Haddock cluster 2	20/89
		2nd Haddock cluster 4	5/89
		3rd Haddock cluster 1	46/89
		4th Haddock cluster 3	13/89
		5th Haddock cluster 5	5/89
wt	E670D	1st Haddock cluster 1	72/94
		2nd Haddock cluster 2	17/94
		3rd Haddock cluster 3	5/94
wt	D674N	1st Haddock cluster 1	83/93
		2nd Haddock cluster 2	10/93

Table S11. Haddock scores [12] for the human EphA2-Sam/SASH1-Sam1 native and mutated complexes. Haddock scores [12] were evaluated as average values over the best 10 structures (IV column) of the specified clusters or just for the best structure (V column) of the cluster. Data are shown for a single cluster, when the best cluster is also the most populated one, or for both the best and the most populated clusters.

EphA2-Sam	SASH1-Sam1	Cluster	Average Haddock Score	Haddock Score
wt	wt	1st (Best & Most populated) Haddock cluster 1	-107 ± 2	-110.0
wt	D661H (HISD)	1st (Best & Most populated) Haddock cluster 1	-96 ± 3	-99.7
wt	D661H (HIS+)	1st (Best) Haddock cluster 2	-92 ± 5	-102.1
		2nd (Most populated) Haddock cluster 1	-89 ± 2	-92.0
wt	E670D	1st (Best & Most populated) Haddock cluster 1	-108 ± 2	-110.6
wt	D674N	1st (Best & Most populated) Haddock cluster 1	-109 ± 1	-111.1

1. (=Reference 27 in the Main Text) Tate, J.G.; Bamford, S.; Jubb, H.C.; Sondka, Z.; Beare, D.M.; Bindal, N.; Boutselakis, H.; Cole, C.G.; Creatore, C.; Dawson, E.; Fish, P.; Harsha, B.; Hathaway, C.; Jupe, S.C.; Kok, C.Y.; Noble, K.; Ponting, L.; Ramshaw, C.C.; Rye, C.E.; Speedy, H.E.; Stefancsik, R.; Thompson, S.L.; Wang, S.; Ward, S.; Campbell, P.J.; Forbes, S.A. COSMIC: the Catalogue Of Somatic Mutations In Cancer. *Nucleic Acids Res.* **2019,** *47*(D1), D941-D947.
2. (=Reference 41 in the Main Text) Ashkenazy, H.; Abadi, S.; Martz, E.; Chay, O.; Mayrose, I.; Pupko, T.; Ben-Tal, N. ConSurf 2016: an improved methodology to estimate and visualize evolutionary conservation in macromolecules. *Nucleic Acids Res.* **2016,** *44*(W1), W344-50.
3. (=Reference 86 in the Main Text) Koradi, R.; Billeter, M.; Wuthrich, K. MOLMOL: a program for display and analysis of macromolecular structures. *J. Mol. Graph.* **1996,** *14*(1), 51-5, 29-32.
4. (=Reference 30 in the Main Text) UniProt Consortium. UniProt: the Universal Protein Knowledgebase in 2023. *Nucleic Acids Res.* **2023,** *51*(D1), D523-D531.
5. (=Reference 33 in the Main Text) Wilkins, M.R.; Gasteiger, E.; Bairoch, A.; Sanchez, J.C.; Williams, K.L.; Appel, R.D.; Hochstrasser, D.F. Protein identification and analysis tools in the ExPASy server. *Methods Mol. Biol.* **1999,** *112*, 531-52.
6. (=Reference 55 in the Main Text) Gasteiger, E.; Hoogland, C.; Gattiker, A.; Duvaud, S.; Wilkins, M.R.; Appel, R.D.; Bairoch, A. Protein Identification and Analysis Tools on the Expasy Server;. In *The Proteomics Protocols Handbook* J.M., W., Ed. Humana Press: 2005; pp 571-607.
7. (=Reference 9 in the Main Text) Ding, Y.; Chen, Q.; Shan, H.; Liu, J.; Lv, C.; Wang, Y.; Yuan, L.; Chen, Y.; Wang, Z.; Yin, Y.; Xiao, K.; Li, J.; Liu, W. SASH1: A Novel Eph Receptor Partner and Insights into SAM-SAM Interactions. *J. Mol. Biol.* **2023,** *435*(19), 168243.
8. (=Reference 53 in the Main Text) Jumper, J.; Evans, R.; Pritzel, A.; Green, T.; Figurnov, M.; Ronneberger, O.; Tunyasuvunakool, K.; Bates, R.; Zidek, A.; Potapenko, A.; Bridgland, A.; Meyer, C.; Kohl, S.A.A.; Ballard, A.J.; Cowie, A.; Romera-Paredes, B.; Nikolov, S.; Jain, R.; Adler, J.; Back, T.; Petersen, S.; Reiman, D.; Clancy, E.; Zielinski, M.; Steinegger, M.; Pacholska, M.; Berghammer, T.; Bodenstein, S.; Silver, D.; Vinyals, O.; Senior, A.W.; Kavukcuoglu, K.; Kohli, P.; Hassabis, D. Highly accurate protein structure prediction with AlphaFold. *Nature* **2021,** *596*(7873), 583-589.
9. (=Reference 54 in the Main Text) Varadi, M.; Anyango, S.; Deshpande, M.; Nair, S.; Natassia, C.; Yordanova, G.; Yuan, D.; Stroe, O.; Wood, G.; Laydon, A.; Zidek, A.; Green, T.; Tunyasuvunakool, K.; Petersen, S.; Jumper, J.; Clancy, E.; Green, R.; Vora, A.; Lutfi, M.; Figurnov, M.; Cowie, A.; Hobbs, N.; Kohli, P.; Kleywegt, G.; Birney, E.; Hassabis, D.; Velankar, S. AlphaFold Protein Structure Database: massively expanding the structural coverage of protein-sequence space with high-accuracy models. *Nucleic Acids Res.* **2022,** *50*(D1), D439-D444.
10. (=Reference 73 in the Main Text) Pucci, F.; Bourgeas, R.; Rooman, M. Predicting protein thermal stability changes upon point mutations using statistical potentials: Introducing HoTMuSiC. *Sci. Rep.* **2016,** *6*, 23257.
11. (=Reference 74 in the Main Text) Pucci, F.; Kwasigroch, J.M.; Rooman, M. Protein Thermal Stability Engineering Using HoTMuSiC. *Methods Mol. Biol.* **2020,** *2112*, 59-73.
12. (=Reference 28 in the Main Text) de Vries, S.J.; van Dijk, M.; Bonvin, A.M. The HADDOCK web server for data-driven biomolecular docking. *Nat. Protoc.* **2010,** *5*(5), 883-97.
13. (=Reference 77 in the Main Text) Xue, L.C.; Rodrigues, J.P.; Kastritis, P.L.; Bonvin, A.M.; Vangone, A. PRODIGY: a web server for predicting the binding affinity of protein-protein complexes. *Bioinformatics* **2016,** *32*(23), 3676-3678.
